# Do young and older adult populations perform equivalently across different automatic face-trait judgements? Evidence for differential impacts of ageing

**DOI:** 10.1371/journal.pone.0322165

**Published:** 2025-05-07

**Authors:** Chithra Kannan, Alex L. Jones, John Towler, Jeremy J. Tree

**Affiliations:** Department of Psychology, Swansea University, Swansea, Wales, United Kingdom; University of Leeds, UNITED KINGDOM OF GREAT BRITAIN AND NORTHERN IRELAND

## Abstract

Accurate implicit personality trait judgements can be made from faces, but as yet the focus has been on young participants making judgements of young faces. The current study sought to explore if similar patterns of performance are seen across the age range, with both young and older adult groups. In addition, we investigated whether implicit trait judgements are associated with cognitive, and trait factors including face recognition, emotional expression perception, autism traits, and alexithymia traits. Across two experiments we explored the extent to which young and older adult populations were able to make accurate implicit associations from faces signalling two different traits – extraversion (positive) and neuroticism (negative). Interestingly, we find that young participants were accurate at making both kinds of automatic trait judgments, and older adults were equivalent to younger controls for the neuroticism personality trait but impaired with automatic extraversion judgements. In both studies, implicit associations were unrelated to any of the other cognitive and trait factors we measured. Based on this pattern of findings, we conclude that face-based implicit trait judgements utilise some independent processes to other face processing abilities, and that the interpretation of particular personality traits is differentially impacted by the ageing process.

## Introduction

Implicit measures are inferred as automatic (spontaneous) and intuitive responses, and consequently are expected to be more robust compared to explicit methodologies [1]. The key implicit measurement tool relating to this literature is the Implicit Association Task (IAT) [2] which is a well-known measure used in evaluating associations underlying implicit attitudes. Using the IAT, Jones et al. have demonstrated that extraversion and agreeableness personality traits can be automatically and accurately inferred using composite facial stimuli [3]. The current study sought to extend this work in a number of key ways. Firstly, using the same IAT paradigm we sought to determine if similar automatic trait judgements can be made from faces with respect to extraversion and the negatively valanced Big-Five trait, neuroticism. Secondly, we also sought to determine whether older and younger participants perform equivalently on such automatic face-trait judgements. Thirdly, we sought to understand the potential cognitive mechanisms that underpin automatic trait judgements from faces, by identifying several key candidates for overlap, including face identity and emotional expression processing.

Despite the extant literature available on facial first impressions, there are only a few studies that investigate both positive and negatively regarded trait judgement mechanisms in the same experiments. Interestingly, several studies have demonstrated that neuroticism personality traits can be accurately interpreted from faces explicitly [4–6]. Other studies exploring trait judgements at zero acquaintance from behavioural information available on online social media show that observers are much worse at assessing neurotic traits compared to extraversion [7,8]. However, no studies as far have considered implicit trait inferences of neuroticism using composite facial stimuli or explored this issue across the age range.

### The impact of age on personality judgements

The apparent age of a face acts as a relevant cue to trait judgements [9,10]. Older adults, in particular, show a positivity bias, evaluating faces as more trustworthy and less hostile, with a bias marked for threatening looking faces or other negative stimuli [11–13]. Furthermore, older adults also tend to perform poorer than younger adults in judgements of criminality [14], aggressiveness [15], and trustworthiness [11]. Overall, these studies have implied that older adults often exhibit poor personality trait judgements. As such, in the current study, we have investigated whether there exists an ‘age-effect’ in processing implicit judgements of extraversion and neuroticism among older adult samples.

Age related cognitive decline is evident from studies investigating face memory, e.g., [16] and face perception, e.g., [17]. Studies investigating the impact of age and face recognition have largely found individuals tend to show better memory for own-age faces [18,19]. This ‘own-age’ bias in face recognition memory testing begs the question whether similar differential patterns may be seen on implicit face-trait judgements, the focus of the current work. In addition to facial memory, it is also well known that there exists an age-related decline in emotion recognition among older adults. A large body of evidence has reported that older adults are often less accurate at identifying emotions from faces in comparison to younger adults, e.g., [20–22], and demonstrate better accuracy for positive emotions than negative emotions [12]. On the contrary, work by Palermo et al. has demonstrated that there was no association between age and emotion perception abilities suggesting that emotion perception is unaffected by age [23].

Overall, it appears that ageing impacts a variety of judgements from faces (i.e., recognition and emotional judgements). In the current study, we sought to establish whether similar age-related effects extend to implicit personality judgements, specifically whether older adults are less accurate than younger adults on our two automatic personality trait judgement tasks. This remains a key unanswered question that is explored by the current study.

### Are automatic face-trait associations linked to face identity and emotional expression recognition?

The most predominantly used theory to explain the cognitive mechanisms underpinning face recognition is the Functional model of face processing [24]. This model suggests that there is possibly an interlink between the encoding of facial identity and trait impressions, and researchers have also debated on whether there exists a dissociation between the two, and that these abilities can be processed independently [25]. Although there is some evidence of a link between facial traits related to an individual face and facial memory, previous studies have implied that face shape, familiarity and subjective ratings of facial memory can affect face recognition abilities [26]. Studies exploring the association between facial memory and trait judgements have associated facial attractiveness [27], trustworthiness and dominance [28,29] with facial memory. Similarly, Bainbridge et al. conducted a study on memorability of face photographs and demonstrated that faces that are perceived to be kind, trustworthy and atypical are often remembered better [30]. Additionally, various other studies exploring individual differences in trait judgements and facial memory have suggested a relationship between facial memory and extraversion trait judgements [9,31], and social anxiety [32]. Furthermore, research also suggests a negative association between facial memory and neuroticism personality traits [32]. Together, these studies therefore appear to demonstrate an association between certain personality traits and facial memory. Nonetheless, it is important to note that these studies have predominantly used explicit methodologies to make personality judgements from facial images. Interestingly, work with individuals who have very poor face recognition ability (i.e., developmental prosopagnosia) indicates preserved explicit trait judgement abilities [25], indicating that trait judgment mechanisms could be independent of face identity recognition.

Work by Knutson suggests that facial expressions of emotion convey information about the target’s internal state and interpersonal information associated with trait inferences [33]. Therefore, personality judgements appear to be an extension of the mechanism involved in processing the emotionality of facial expressions. Previously it has been implied that emotional expressions can communicate behavioural intentions [34]. Particularly, faces that are evaluated negatively may contain subtle cues that resemble angry expressions, and faces evaluated positively may contain cues to happy expressions. For example, work by Todorov suggests that subtle cues that represent emotions even from neutral stimuli can contain information about an individual’s personality [35].

The evidence considered here indicates that there may well be overlap between functional processes linked to emotion processing and particular trait judgements in the general population – however, two key dimensions linked to poor emotion processing have been identified that are relevant for consideration: namely, autism and alexithymia. Populations with higher levels of autism (even sub-clinically) have been reported to be poor at emotion processing [36,37]; so, might such individuals also do poorly on trait judgements? While studies have reported an association for individuals with autism like traits and performance on measures of facial memory and emotions, few studies have considered relative performances on face-trait judgements among such individuals. Researchers have indicated that individuals with autism spectrum disorders (ASD) show normal trait judgements for dominance and trustworthiness [38,39]. On the contrary, work by Adolphs et al. has reported that individuals with ASD tend to rate untrustworthy faces to be highly trustworthy [40]. Although, individuals with autism exhibit impairment in emotion recognition and show reduced cognitive empathy, these poor inferences of facial expression perception are mainly predicted by a co-occurring condition termed as alexithymia in individuals with autism [41–43]. Work by Brewer et al. have implied that in the face processing system, emotion detection could take place before personality trait inferences, and therefore individuals with alexithymia could possess selective impairment at detecting emotions [44]. As a consequence, in the current study of automatic face trait judgement, we sought to include measures of both autism and alexithymia as a means of determining if these variables have an impact on individual performance.

### The current study

Taken together, based on the above literature, we undertook two behavioural studies measuring implicit personality judgements across young and older adult populations. Given it is already established that young participants can reliably make implicit trait judgements with the extraversion personality trait [3], in Experiment 1a, we sought to both replicate this finding with younger participants and establish the pattern seen with older adults. In addition, we sought to examine whether performance in this context is related to other cognitive and behavioural variables: which included, autism traits, alexithymia traits, face recognition and face emotion perception. In Experiment 1b, we extend this investigation to a more negatively regarded trait among the Big-Five – namely, *neuroticism* across ages, a trait as yet not investigated in this context. As in Experiment 1a, we sought to determine the potential impact of ageing on such judgements and whether IAT performance was impacted by the other cognitive and behavioural factors mentioned above. In addition, we sought to determine whether self-rated levels of neuroticism predicted IAT performance - in order to explore whether individuals with higher levels of neuroticism may be better at perceiving the same traits they possess in others.

## Materials and methods

### Ethics statement

Ethical approval was obtained from Swansea university department of psychology ethics committee. All the tasks and questionnaires used in this study were designed using the software Gorilla [45]. All participants provided informed written consent before taking part in the study and were fully debriefed after study completion.

### Participants

Using a between group design, power analysis was conducted on effect size estimates using G* power [46] with a minimum *n *= 70 in each study, with α* *= .05, *β *= .80 and expected a conventionally medium effect size *d* > .3. We have also included Bayesian effect size distributions for null hypothesis testing and power estimates [47,48]. The younger adult population (Age range 18–35) were recruited using Swansea university participant pool for psychology course credits where the students came to Swansea University face lab to complete the experiments; and through Prolific.ac (online platform), where participants were paid £3 for participation. The older adults were recruited through the Swansea older adult volunteer participant panel (Age range 55 above). All the older adults came to the Face research Swansea lab to take part in the study. Only Caucasian participants were included in these studies to avoid other-ethnicity effects [49]. No participant was excluded from the study after considering the improved scoring algorithm outlined by Greenwald et al. [50]. The recruitment period for this study was between 20/02/2018–30/09/2019.

#### Extraversion IAT. .

In Experiment 1a, there were 118 young adults (age *M = *23.50, *SD = *4.79; 68 females), and 62 older adults (age *M = *69.76, *SD = *7.17; 32 females), with a total sample of *n *= 180.

#### Neuroticism IAT. .

In Experiment 1b, there were 120 young adults (age *M = *24.58, *SD = *4.89; 71 females), and 50 older adults (age *M = *67.96, *SD = *8.82; 31 females), with a total sample of *n *= 170.

### Measures

***Autism Spectrum Quotient* (AQ).** A 50- item AQ scale [51] was used to measure autistic traits. This measure includes 5 subscales made up of 10 questions each measuring: *social skill, attention switching, attention to detail, communication* and *imagination.* Participants recorded their responses using 4-point Likert scales ranging from 1 = ‘definitely agree’ to 4 = ‘definitely disagree’. Participants with a total score above 32 was considered as individuals with high AQ.

***Toronto Alexithymia Scale*** (TAS-20) [52] was used as a screening measure to assess Alexithymia traits. The TAS measures three main facets of Alexithymia: difficulty identifying feelings, difficulty communicating or describing emotions to others, externally oriented style of thinking. Participants completed the questionnaire using 5-point Likert scales ranging from 1 = ‘strongly disagree’ to 5 = ‘strongly agree’. Individuals with total score above 61 were classified as individuals with high alexithymia, and a score below 51 was considered as low alexithymia.

***Mini- International Personality item pool*** (IPIP) [53]. This 20-item scale measures traits related to the Big Five personality (Openness, conscientiousness, extraversion, agreeableness, and neuroticism) with 4 items per trait. This self-report measure was used to identify whether there was a relationship between self-perception of neuroticism score and the neuroticism IAT.

***Face Trait Implicit Association Task (IAT).*** A novel version of the IAT [2] was used in this study with female composite facial stimuli.

***Stimuli.*** Two sets facial composites were generated from a sample of 64 Caucasian females (age *M* = 21.03, *SD* = 1.94) who completed the 20-item measure of mini-IPIP from the Big-Five personality inventory [53]. The photographs were averaged using psychomorph. These images were obtained from previous study by Kramer and Ward [4], and for our studies we created novel versions of the facial composites.

**Fig 1:**
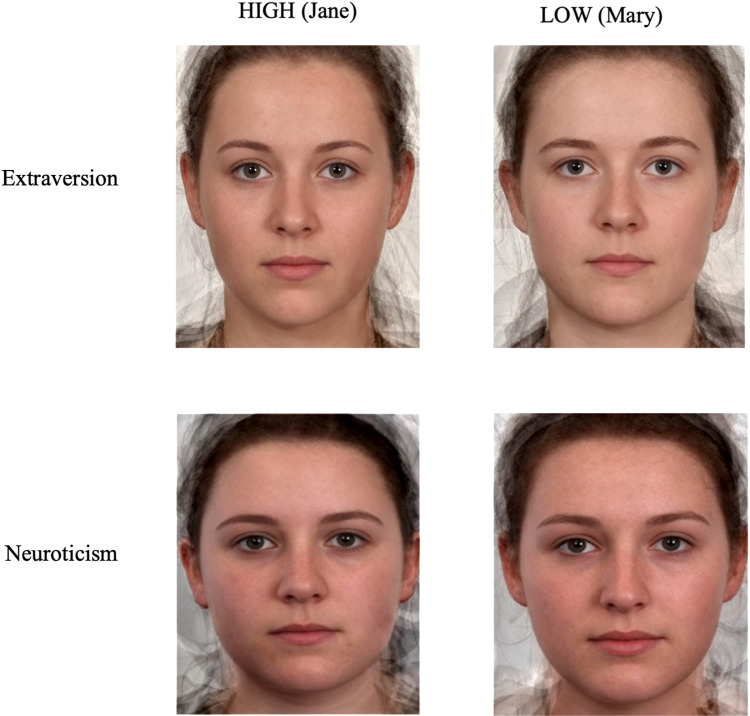
Facial composite stimuli employed in the IAT. Note: High-level trait composite faces ‘Jane’ appears on the left and low-level trait composite faces ‘Mary’ appear on the right.

Although previous studies have investigated on personality traits using both male and female composites, the current study only explored implicit trait judgements using female composites. Arguably female composites are suggested to consist of cues to personality that can be measured efficiently compared to male composites, as male composites have been reported to contain fewer cues to their actual personality, e.g., [5]. As such, for the purposes of this study, we included female composite facial stimuli portraying high extroversion, low extroversion, high neuroticism and low neuroticism personality traits (See Fig 1) and words describing personality traits high extraversion (Confident, Sociable, Outgoing, Talkative), low extraversion (Shy, Quiet, Reserved, Thoughtful); high neuroticism (Moody, Vulnerable, Insecure, Worrying) and low neuroticism (Hardy, Relaxed, Secure, Calm).

***Procedure.*** Across all studies, high extroversion and high neuroticism composites were called ‘Jane’, the low composite faces were called ‘Mary’. In this task, keyboard response ‘E’ was used for categories on the left and ‘I’ was used for the categories on the right. The task began with a general instruction and familiarization phase where words describing personality traits were presented. Followed by a screen presenting the categories of composite faces labelled Jane and Mary for a fixed duration of two minutes at a larger resolution of 415 x 495. Participants were instructed to familiarize with the words and faces in order to sort them in the following blocks. Instructions were given before each block.

**Fig 2:**
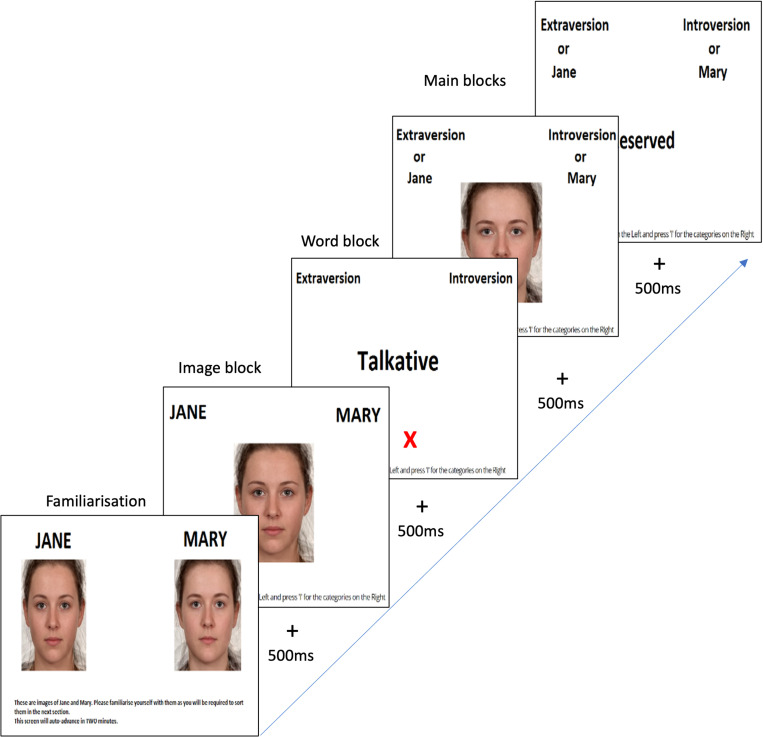
Extraversion IAT block design. *Note*: This figure shows an example of block design employed in the extraversion IAT. A fixation cross was presented for 500ms between trials. A red cross appeared when participant made incorrect responses. Participants were then allowed to make the correct response before moving to the next trial. Reaction times were recorded from image presentation until correct response.

Following the familiarization task, a practice block was presented with instructions to sort images of Mary and Jane. Participants completed 7 blocks of the IAT based on the standard block design described by Greenwald et al. [50] (See Table 1). In our tasks, a congruent block was defined as response key assigned to a high trait face (Jane) to the same key as high trait words. An incongruent block was defined as response key assigned to a high trait face (Jane) when paired with same key as low trait word categories. For the first 4 blocks, Mary appeared on the right and Jane appeared on the left. The reason behind this structure of the task is to familiarize participants with the images under usual response conditions. After the first 4 blocks, the position of Mary and Jane switched. To counterbalance the conditions, half the participants started with the congruent then incongruent version and the other half vice versa. When participants made an error in their response, a red cross appeared on the screen below the stimulus presented (See Fig 2 for IAT block design). After this, participants were allowed to correct the response by entering the correct keyboard response. Based on the scoring algorithm [50], any participant scoring below 300ms or over 10000ms were removed, latencies from these four trials were converted into D scores (see Table 2 for IAT scoring algorithm). A fixation point for 200ms was presented between each trial. Reaction times were measured, and accuracy was determined by whether there was a bias towards congruent over incongruent face-word association.

**Table 1:** IAT block design [50].

Block	No. of trials	Function	Item assigned to left key	Items assigned to right key
1	20	Practice	Jane images	Mary images
2	20	Practice	Extraverted words	Introverted words
3	40	Test	Jane images + extraverted words	Mary images + introverted words
4	40	Test	Jane images + extraverted words	Mary images + introverted words
5	20	Practice	Mary images	Jane images
6	40	Test	Mary images + extraverted words	Jane images + introverted words
7	40	Test	Mary images + extraverted words	Jane images + introverted words

*Note:* This table is an example of the congruent conditions of the IAT. Blocks 5,2,6,7 appear at the start in the incongruent conditions followed by blocks 1,3,4. This is an outline of the extraversion IAT block design. Identical design was used for neuroticism IAT with words and images for the trait.

**Table 2:** IAT D score calculation based on the improved IAT scoring algorithm.

Step	Improved Algorithm
1	Use data from B3, B4, B6, & B7
2	Eliminate trials with latencies > 10,000ms; eliminate subjects for whom more than 10% of trials have latency less than 300ms
3	Use all trials
4	No extreme-value treatment (beyond Step 2)
5	Compute mean of correct latencies for each block
6	Compute one pooled SD for all trials in B3 & B6; another for B4 & B7
7	Replace each error latency with block mean (computed in Step 5) + 600 ms
8	No transformation
9	Average the resulting values for each of the four blocks
10	Compute two differences: B6 - B3 and B7 - B4
11	Divide each difference by its associated pooled trials SD from Step 6
12	Average the two quotients from Step 11

***IAT scoring procedure -*** The IAT measures the extent to which implicit automatic associations can be made. The IAT effect is based on reaction times obtained from four trials comparing the congruent and incongruent blocks. Since a correct response was required after an incorrect response was made, instead of replacing each error latency with block mean as described in step 7 from the scoring algorithm [50], the additional time taken to correct the response was added to the initial reaction time as error latency [3] error trials were included in the analysis by including latencies between stimulus presentation and correct response which is a built-in error penalty [3,7].

***The Cambridge Face Memory task (CFMT)- upright version.*** The CFMT (Duchaine & Nakayama, 2006) was used to test face memory. Images were obtained from the original study by Duchaine and Nakayama [54]. A target image was presented with two distractor images. The CFMT task presentation is made up of four stages: stage 1- Practice task, stage 2 - Introduction/same images, stage 3- novel images, stage 4 - novel images with noise. Participants completed this task using standard procedures, (see [54]). A total accuracy score was calculated from the three test blocks with a maximum possible score of 72.

**Fig 3:**
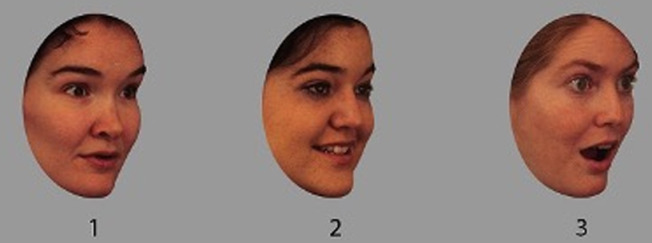
Emotion matching task. *Note:* showing an example of the emotion matching task in right profile angle with face portraying happiness and surprise emotions - the correct ‘odd one out’ response here would be 3. *KDEF image ID 050Hap&Sur01* [56].

***Emotion matching task*** (100-item matching task) [55]. The images used in this study were obtained from Karolinska Directed Emotional Faces Database (KDEF) [56]. This comprised of full colour images portraying six basic emotions (fear, anger, disgust, happy, surprise and sad) in either front profile, left profile or right profile angles. Participants were presented with 5 blocks of 20 trials each. Within each trial, there were three images depicting facial expression. Each target face appeared in a triad with two other distractor faces. The faces portrayed two similar emotion and one different emotion that is commonly confused with the other emotion. The images used emotions such as fear, anger, disgust, happy, surprise and sad. The images were paired with emotions that are commonly confused (See Fig 3 for example). This study followed the standard procedure used in Palermo et al. [55]. Accuracy for each block was calculated with a maximum possible score of 100.

We included only a single procedural difference across experiments, in these we did not include a time limit on the emotion matching task for the older adult groups. In the original task, a 4500ms timer was included for image presentation after which the image disappears and an additional time window of 7000ms to make responses. If the participants were unable to make responses within this time frame, the response is encoded as “timed out”. Several researchers have demonstrated that older adults take longer to process such information compared to younger adults (e.g., [13,14]). Initially we conducted a pilot study examining whether older adults are able to complete the task efficiently with the timer but unfortunately several blocks within the task were timed out for older adults. Hence, for better quality of results, we have excluded the timer in the emotion matching task for older adults.

## Experiment 1a: Extraversion trait judgements across ages

In Experiment 1a, we sought to measure whether young and older adults are able to make implicit extraversion face-trait judgements using young adult composite facial stimuli. Further, it was investigated whether such associations are predicted by age, autism traits, alexithymia traits, facial memory and emotion perception in non-clinical young adult populations. See Table 3 for descriptive statistics.

**Table 3:** Descriptive statistics for tasks and measures in experiment 1a.

Extraversion		N	Minimum	Maximum	Mean	Std. Deviation
**Age**	Young	118	18	35	23.50	4.79
	Old	62	57	89	69.76	7.17
**IAT D score**	Young	118	-.787	.971	.125	.353
	Old	62	-1.028	1.223	0.0003	0.48
**AQ**	Young	118	4	45	19.09	8.46
	Old	62	7	36	17.484	7.23
**TAS20**	Young	118	24	89	50.27	12.45
	Old	62	23	88	44.565	11.92
**CFMT**	Young	118	34	72	53.18	9.50
	Old	62	24	72	47.968	11.30
**Emotion task**	Young	118	42	86	67.86	7.42
	Old	62	42	90	70.855	8.49

### Results

The reaction time data obtained from the IATs were converted into IAT D scores using Python code following the scoring algorithm (Greenwald et al., 2003). The raw data files and Python scripts used to produce the results of this study are available on the open science framework (https://osf.io/4nvgw/?view_only=8d92e7ee4c484f7f96773010713db499). Where data did not meet assumptions for a parametric test, a non-parametric equivalent was used for statistical analysis.

#### Implicit Extraversion personality trait judgments from faces.

A one sample *t*-test against chance (zero) was conducted to identify whether there was a significant relationship between faces and personality trait words. The results revealed that the *young adults* were able to make accurate implicit personality trait judgements from faces for Extraversion, *IAT D *= .13 (*SD *= .35), 95% CI [.06,.19] *t* (117) = 3.85, *p *< . 001, Cohen’s *d *= .354. Young adults were faster and more accurate on trials where highly extraverted faces were paired with highly extraverted words, and on trials where lower trait faces were paired with lower trait words, supported by positive *IAT D* score.

On the contrary, the *older adults*’ sample did not significantly produce an IAT effect, *IAT D *= .0003 (*SD *= .48), 95% CI [-.12,.12] *t* (61) =.006, *p *= .996, Cohen’s *d* = .13. It is interesting to note that the IAT D score is approximately zero, denoting that there is not much difference in response latency between conditions (congruent and incongruent). To further explore the null hypothesis, we considered a Bayesian approach. A Bayesian one sample *t*-test revealed that there was moderate evidence for the null hypothesis H_0_ with BF_01_ = 7.190, suggesting that these results support the acceptance of the null hypothesis.

**Fig 4:**
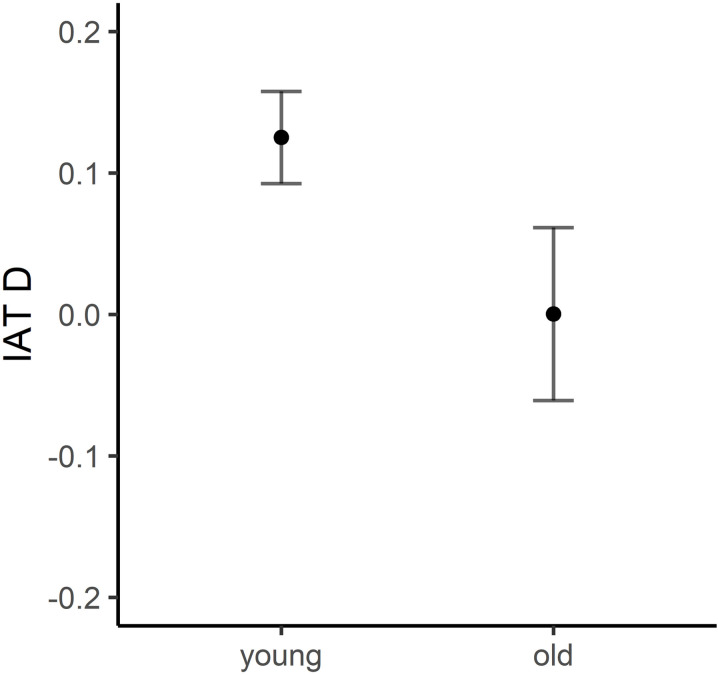
Extraversion IAT group differences between young and older adult samples. *Note.* Black dots showing average extraversion IAT D for young and older adult groups and error bars representing standard error.

A two-tailed independent sample *t*-test was conducted to determine whether there were differences in the performances of young and older adult samples in identifying extraversion implicit personality trait judgements from faces using the IAT. A significant group difference was found between young and older adult participants in the IAT (*t*(178) = 1.98, *p *< .05, Cohen’s *d* = .31). The findings of this study revealed that young adults were better at associating extraversion images and extraversion trait words compared to the older adult sample (See Fig 4).

#### Relationship between Extraversion IAT, age, autism traits, alexithymia, face memory and emotion perception.

An important aspect was to determine whether implicit extraversion face-trait judgements was independent of other cognitive and trait factors. Firstly, correlational analyses was conducted to explore the relationships between implicit judgements of Extraversion (IAT), age, autism traits (AQ and subscales), alexithymia traits (TAS20 and subscales), facial memory (CFMT) and emotion perception (Emotion matching task). For the emotion matching task, given the differences in the methodology for younger and older adults, we have reported separate correlations for young adults and older adults if this measure correlated with any of the factors.

There was a negative association between extraversion IAT and age (*rs* (178) = -.165, *p *< .05), where accuracy for extraversion (IAT) implicit associations decreased with age. All other cognitive and behavioural measures produced non-significant correlations against extraversion IAT. There was a negative association between age and face memory (*rs* (178) = -.245, *p* < .001), where young adults perform better in tasks related to facial memory compared to older adults. This finding is also supported by an independent *t*-tes*t* where age related cognitive decline is reported for facial memory (*t* (178) = 3.27, *p* = .001, *d* = .51). There were no significant differences be*t*ween young and older adults (*p* > .05) performance on the emotion matching task. However, older adults (average latency *M *= 4496.162, *SD *= 1614.680) significantly take longer than young adults (average latency *M *= 2081.40, *SD *= 410.10) in emotion perception abilities (*t* (175) = 15.33, *p* < .001, *d *= 2.44).

In line with the previous literature (e.g., Cook et al., 2013), there was a positive association between autism and alexithymia scales (*rs* (178) =.520, *p *< .001). As demonstrated in previous work [55], there was a positive association between facial memory and emotion perception for both groups, young adults (*rs* (117) =.338, *p < *.001), older adults (*r* (60) =.323, *p* < .05). All other correlations were non-significant after applying Bonferroni corrections and the pattern was identical when considering Bayesian correlations (See S1 Table).

*Secondly,* a multiple linear regression analyses was conducted to explore whether implicit extraversion trait judgements can be predicted by age, autism quotient, alexithymia quotient, facial memory or emotion perception among younger and older adult samples. Firstly, we considered whether age was a significant predictor for extraversion trait judgements performance on the IAT for young and older adults. The results indicated that (*F* (1, 178) = 5.299, *p* < .05, *R*^2^ = .029), age significantly predicts extraversion implicit associations (*β *= -.17, *p < *.05). Secondly, we conducted two separate regressions for young group against other factors, and older adults’ group versus other factors. Considering the differences in the methodology for the emotion task employed by older adult participants, here we have conducted regressions for the groups separately. As such the results indicated that that was no significant effect between extraversion IAT and AQ, TAS, CFMT and Emotion task across both groups (See Table 4 for detailed results of the regression).

**Table 4:** Multiple linear regression analysis for extraversion IAT.

	t	p	β	F	df	*p*
**IAT**						
Model				5.299	1,178	.022
Age	-2.30	.022	-.170			
**Young adults**						
Model				2.272	4,113	.066
AQ	-.417	.677	-.041			
TAS20	-1.700	.092	-.170			
CFMT	-1.581	.117	-.153			
Emotion task	-1.594	.114	-.153			
**Older adults**						
Model				1.202	4,57	.320
AQ	-.137	.891	-.023			
TAS20	-.810	.421	-.135			
CFMT	1.499	.139	.203			
Emotion task	.467	.642	.065			

## Experiment 1b: Neuroticism trait judgements across ages

In Experiment 1b, we seek to identify whether implicit associations of *neuroticism*, a negatively regarded trait among the big-five, can be made from composite facial stimuli across ages. As before, we also sought to further explore whether implicit judgements of neuroticism personality traits can be predicted by individual traits of autism and alexithymia, self-perception of neuroticism, facial memory and emotion perception across young and older adult groups. See Table 5 for descriptive statistics.

**Table 5:** Descriptive statistics for tasks and measures in experiment 1b.

Neuroticism		N	Minimum	Maximum	Mean	Std. Deviation
**Age**	Young	120	18	35	23.50	4.79
	Old	50	55	89	67.96	8.822
**IAT D score**	Young	120	-.787	.971	.125	.353
	Old	50	-.596	1.150	.14	.422
**AQ**	Young	120	4	45	19.09	8.46
	Old	50	6	33	14.94	6.988
**TAS20**	Young	120	24	89	50.27	12.45
	Old	50	22	75	42.68	11.790
**CFMT**	Young	120	34	72	53.18	9.50
	Old	50	22	71	48	10.654
**Emotion task**	Young	120	42	86	67.86	7.42
	Old	50	37	85	69.76	9.417
**Mini-** **IPIP**	Young	120	4	20	12.80	3.55
	Old	50	4	16	9.78	3.112

### Results

#### Implicit Neuroticism personality trait judgments from faces.

Analysis procedure was identical to experiment 1b. The results of the one sample *t*-test revealed that the young adults (*Neuroticism IAT D *= 0.16 (*SD *= 0.39), 95% CI [.093,.235] *t* (119) = 4.59, *p *< .001, *d *= .42) and older adults (*Neuroticism IAT D *= .14 (*SD *= 0.42), 95% CI [.020,.260] *t* (49) = 2.346, *p *= .023, *d *= .33) were able *t*o make accurate implicit personality trait judgements from faces for Neuroticism. Participants were faster and more accurate on trials where high neuroticism faces were paired with high neuroticism words and on trials where lower trait faces were paired with lower trait words. These results are the first to reveal that neuroticism personality traits can also be judged accurately and implicitly from composite facial structure across ages.

**Fig 5:**
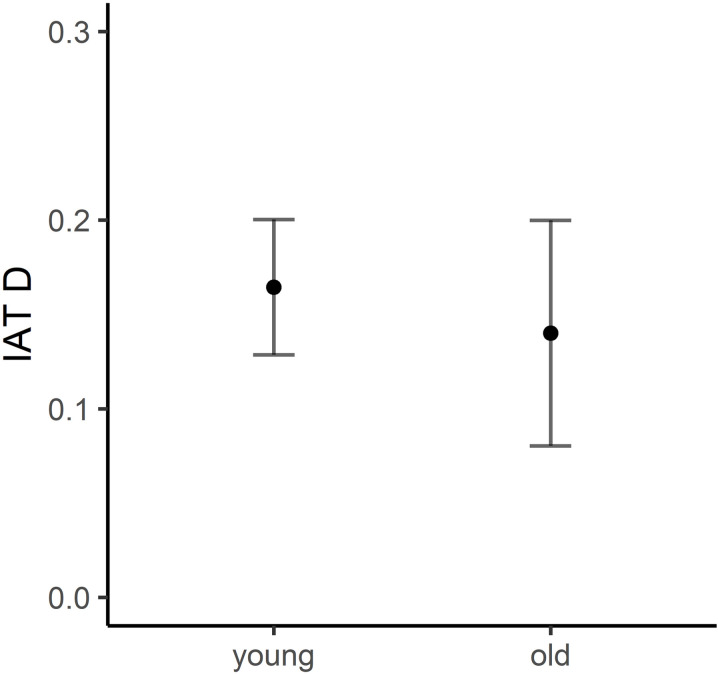
Neuroticism IAT group differences for Young and Older adult samples. *Note.* Black dots showing average neuroticism IAT D for young and older adult groups and error bars representing standard error.

An independent sample *t*-test was conducted to determine whether there were differences in the relative performances of the two age group samples on our neuroticism IAT task (See Fig 5). There were no significant group differences between young and older adults’ performance on the neuroticism IAT (*p* > .05). Furthermore, a Bayesian *t*-*t*est revealed that there was moderate evidence for the null hypothesis H_0_ with BF_01_ = 5.23, suggesting that these results support the acceptance of the null hypothesis.

#### Relationship between Neuroticism IAT, age, autism traits, alexithymia, self-perception of neuroticism, face memory and emotion perception.

As before, here we sought to determine whether implicit neuroticism face-trait judgements was independent of other cognitive and trait factors. A correlational analysis was conducted to explore the relationship between implicit Neuroticism trait judgements, age, autism traits, alexithymia traits, self-perception of neuroticism, facial memory and emotion perception. Similar to Experiment 1, here we have reported separate correlations for young adults and older adults’ emotion matching task if this measure correlated with any of the factors. Neuroticism IAT produced non-significant correlations against other cognitive and behavioural factors. Similarly, in accordance with previous work, e.g., [9], self-perception of neuroticism was unrelated to implicit judgements of neuroticism.

There was a negative association between Age and AQ-subscale attention switch (*rs* = -.255, *p* < .001), where difficulties in attention switching were associated with lower age. Similarly, there was a negative association between age and alexithymia traits (*rs* (168) = -.249, *p* = .001), where young adults appear to report more alexithymia like traits compared to older adults. There was a negative association between age and self-perception neuroticism (*rs* (168) = -.235, *p* = .002), where young adults self-reported to have more neuroticism traits in comparison with older adults. However, the means of both groups are on the lower band of these scales (See Table 5: descriptive statistics).

There was a positive association between autism and alexithymia scales (*rs (*168) = 0.387, *p *< .001; Cook et al., 2013). Similarly, there was a positive association between autism and self- perception neuroticism scales (*rs* (168) = 0.276, *p *< .001). A positive association between alexithymia traits and self-perception neuroticism scales (rs (168) = 0.294, *p* < .001), specifically TAS-subscale describing feelings (*rs* (168) =.238, *p* = .002) and identifying feelings (*rs* (168) =.413, *p* < .001). Several studies have predominantly associated alexithymia and neuroticism self-report measures and these studies have largely suggested that both these measures evaluate emotional distress and hence tend to be highly correlated (See [57,58]). As demonstrated in previous work [55], there was a positive association between facial memory and emotion perception among the older adult group (*rs* (48) =.341, *p* = .02). All other correlations in this experiment were non-significant after applying Bonferroni corrections and considering Bayesian correlations (See S2 Table).

Identical to the findings reported in Experiment 1a, older adults were significantly poorer at face memory compared to young adults (*t* (159) = 3.174, *p *= .002, *d *= .541); and there were no significant group differences in the emotion matching task (*p *> .05) for young and older adults. However, older adults (average latency *M *= 4311.756, *SD *= 1486.13) are significantly slower in responses than young adults (average latency *M *= 1898.76, *SD *= 560.37) groups in emotion perception abilities (*t* (168) = 15.40, *p* < .001, *d *= 2.59).

A multiple linear regression analysis indicated that there was no significant effect between neuroticism IAT and other factors (See Table 6). In sum, these findings appear to indicate that the ability to identify neuroticism personality from faces is independent of age, autism traits, alexithymia traits, self-perception of neuroticism, facial memory and perception of facial expression. Although the regression model for young adults’ neuroticism IAT was non-significant, there is a small significant facial memory predictor. It appears from the result that facial memory negatively influences neuroticism trait judgement. All VIF scores for the variables were below 2, suggesting there was no multicollinearity in the data. However, there was no correlation between facial memory and neuroticism IAT and hence it is possible that this predictor is a result of false positive (Type 1 error) [59] and could be disregarded. Moreover, overall accuracy score on the CFMT of the sample is low compared to other groups. Thus, it is possible that such effects could imply that low scorers on the CFMT are still intact with personality associations. Furthermore, if such results are possible, it could further suggest that individuals with face processing deficits (such as Developmental Prosopagnosics) might be able to form accurate neuroticism personality judgements. Regardless of this, it is highly possible that this effect between the CFMT and the IAT is occurring due to chance.

**Table 6:** Multiple linear regression analysis for neuroticism IAT.

	t	P	β	F	df	p
**IAT**						
Model				0.001	1,168	0.97
Age	-.033	0.97	-0.003			
**Young**						
Model				2.013	5, 114	0.082
AQ	-1.854	0.066	-0.181			
TAS20	0.811	0.419	0.081			
Self-perception	0.631	0.530	0.060			
CFMT	-2.201	0.030	-0.211			
Emotion task	0.715	0.476	0.070			
**Old**						
Model				0.236	5, 44	0.945
AQ	0.967	0.339	0.164			
TAS20	-0.204	0.839	-0.035			
Self-perception	-0.253	0.801	-0.042			
CFMT	0.123	0.903	0.020			
Emotion task	-0.503	0.617	-0.080			

## General discussion

It is evident that trait judgements are signalled from faces with limited previous interaction [7,60], even when these judgements are implicit associations [3]. Currently it is not clear what factors, if any, determine the degree of accuracy for such implicit personality trait judgements. This begs the question, might such judgements be impacted by the ageing process, or overlap with particular cognitive processes? We have established, using images of young female composite facial stimuli, that young adults are able to form accurate implicit associations for extraversion (replicating [3]). This accurate implicit trait judgement ability also extends to the trait of *neuroticism,* an entirely novel finding. Interestingly, our testing of older adults indicates that their performance was equivalently accurate for neuroticism judgements, but not so for extraversion, which may indicate that the ageing process attenuates successful implicit trait judgements to a varying degree across trait types. However, in neither of these studies was judgement performance related to a number of other variables we measured – including, autism traits, alexithymia traits, self-perception of neuroticism (for neuroticism IAT), facial memory or emotion perception. In short, the evidence suggests that the mechanisms underpinning such automatic trait judgements are likely independent from these other variables and processes.

People tend to be more proficient and show better accuracy for recognizing individuals from own-group compared to other-groups [49,61,62]. Additionally, previous reports suggest that there exists a positivity bias among older adults when interpreting trustworthiness traits (e.g., [14]). Given in the current study, older adults can form accurate judgements for neuroticism but not extraversion from young facial stimuli, a general own-age bias effect cannot explain the different patterns of performance. Furthermore, facial memory was unrelated to implicit personality judgements across ages (for extraversion and neuroticism). As such, own-age bias or positivity-bias does not explain the findings reported across our studies. However, it remains unclear whether this pattern reflects the possibility that the ‘signal’ generated by young face images is to some extent harder for older adults to detect in one case (extraversion) but not another (neuroticism). That is, it remains to be determined whether a similar pattern might be seen if we had utilised older adult composite images with our older participant sample (we will discuss this further in the limitations section).

Nonetheless, in relation to facial memory, negative traits are known to be more memorable from faces [29,63]. Negative traits might signal facial cues that communicate potential threat [64]. For example, Zebrowitz et al. [13] has suggested that traits such as hostility and competence can be clearly communicated from young facial stimuli and variations in health and aggressiveness from older facial stimuli. We might therefore speculate that a reason for the variability of the findings presented across both our studies may centre on cue availability from faces for positive and negative traits that underlie social perceptions.

Our current work is also consistent with previous literature demonstrating that self-perception of neuroticism is nevertheless unrelated to perceivers associations of face-trait judgements (e.g., [9]). More generally, it is apparent that neuroticism personality trait characteristics involve experiencing negative affect and anxiousness (e.g., [65]. Given that neuroticism is a negatively regarded trait, and its characteristic description involves traits such as anxiousness, and specific difficulties with social interaction, evidence suggests that this trait is highly correlated with autism traits [66]. However, our work indicates the ability to make implicit neuroticism trait judgements is unrelated to autistic traits. On balance then, it seems that although our evidence indicates the neuroticism trait can be detected from faces across both age groups, it seems unlikely this is related to the traits of the ‘perceiver’ and more likely signalled from the ‘perceived’. Our work indicates that a focus of research attention may be needed around the processes of social interaction, in that individuals with high neuroticism traits may be perceived differently from facial structure alone.

Consistent with previous literature, older adults performed poorly on tasks related to facial memory compared to young adults. Implying that there is an age-related decline in facial memory (e.g., [67]). In addition to facial memory, it is also well known that there exists an age-related decline in emotion recognition among older adults (e.g., [20–22]). Our findings revealed that older adults show normal emotion recognition abilities in time unconstrained conditions, i.e., older adults show equivalent accuracy performance when provided more time to do so (e.g., [23]). However, older adults are significantly slower than young adults in perceiving emotions. Facial expressions fluctuate rapidly and dynamically in social interactions, and therefore the emotion perception abilities among older adults are reported with some caution. The high accuracy in the task is reflective of longer time duration affecting expression perception abilities, and thus, the possibility of atypicality cannot be disregarded. Previous research has indicated that when response times are not provided, it is possible that the high accuracy in such tasks that appear to indicate normal performance may be overshadowed by the application of successful, but abnormal facial feature matching strategies [54,68,69]. Although aging impacts a variety of judgements from faces such as facial memory and emotion perception, the current evidence indicates that both appear to be independent of any age-related effects on personality judgements – and in fact the pattern may be further complicated by the particular trait under scrutiny.

Empirical studies have associated personality judgements and individuals with differences in social perception [33,70]. Despite research suggesting that there might be an association between facial memory and extraversion personality trait judgements [9], we did not find any such associations. Similarly, for expression perception, previously it has been suggested that personality judgements are an extension of the mechanism involved in processing the emotionality of facial expressions [33,71]. However, we did not find any such associations between implicit trait judgements and expression perception. Research suggesting a relationship between traits and emotions have largely been conducted on trustworthiness judgements. As such the mechanisms underpinning trait judgements may be differentially impacted by expression perception based on the trait being judged. However, it is not yet widely understood whether such associations extend to other types of personality traits, in this case, the big-five dimensions.

With regard to face processing models [24,72], we suggest that faces contain structural similarities that are shared with emotions and face identity. There may be some shared aspects of face recognition at the early stages of visual perception that utilise similar processes to infer emotions, identity and traits; these perceptual processes can further differentiate for trait-specific processes independent of emotion and identity. Although the shared routes can be used to extract information regarding a specific process (e.g., emotion, identity, traits), information specific processes can be extracted regardless of whether other aspects of recognition are being processed or not. For example, information regarding personality can be processed regardless of whether information regarding emotions is present in the face. Thus, it is possible for trait judgements to utilise some independent mechanisms as opposed to a shared route. We further imply that there may be potential differences in the mechanisms underpinning trait judgements based on the nature of the trait itself (positive or negative). Since negative traits can communicate increased level of threat, we suggest that traits such as neuroticism can be universally recognized across ages. Furthermore, another interesting population to consider would be individuals with face processing deficits. Researchers have suggested that the neural mechanisms underpinning trait impressions and face identity recognition are dissociable [25]. Hence, we further speculate that DP candidates could possibly be able to make accurate implicit personality judgements of neuroticism personality traits. Although there is some evidence for intact extraversion and trustworthiness trait judgements among the DP candidates (e.g., [25,73]); there is yet a dearth of evidence for this independent process among the DP candidates. Overall, we conclude that implicit trait judgements are likely processed independently in the cognitive system in comparison with other perceptual processes.

### Limitations and implications

Although the emotion perception task employed for older adults did not include a time limit (based on findings from the initial pilot testing of the task), the reaction times that are reported are significantly longer for older adults compared to young adults. Thus, in future, creating measures to increase the time latency and including a timer for these groups would aid in establishing whether there is atypicality present among older adult groups. We also suggest future studies to replicate the findings of older adults using a larger sample size.

It is clear that our older adult participants were not as accurate as younger participants at making implicit extraversion trait judgements from faces. However, it remains unclear whether this is because of an issue with perception of this trait from the face (that is younger faces provide a poorer ‘signal’ of this trait to older adult ‘perceivers’ – akin to an own age ‘bias’) or emerges as a consequence of some impact of ageing on the process that underpins such trait judgements. But for this latter possibility to be true, we would also have to assume that ageing impacts this process such that it has differential consequences across different trait judgements (i.e., neuroticism more so than extraversion). We suggest to further explore this issue by testing older and younger participants with both young and older adult face composites linked to the extraversion trait. Clearly, if the poorer performance for older adults is in some manner akin to an age ‘bias’, then we would expect the current age effect pattern to reverse with older adult face stimuli.

Given that, neuroticism personality traits were accurately inferred from faces across different age groups, it would add to theoretical frameworks to better understand the mechanisms involved in processing negative trait judgements using other-ethnicity samples and developmental prosopagnosia samples. We further imply that exploring the effects of neurological mechanisms underpinning implicit trait judgements for positive and negative traits would add to theoretical frameworks that capture in face trait judgements; and to study the brain regions associated with positive and negative trait inferences using fMRI and the IAT design employed within our studies (specifically extraversion and neuroticism) to better understand the neural frameworks underpinning trait judgements.

## Conclusion

In sum, the findings of the current work revealed that young adults are able to make accurate implicit face-trait judgements of extraversion and neuroticism. This latter finding is entirely novel and opens the door to future work that may wish to explore these contrasting traits. Interestingly, older adults were only able to make accurate implicit judgements for neuroticism (a negatively regarded trait). As a consequence, it appears that ageing can impact such trait judgements, but not in all cases. We will seek to investigate these contrasting ageing effects in future work. Finally, our work indicates that the processes underpinning such implicit personality judgements are not associated with ability linked to other face related processing systems (such as emotion/identity recognition) or factors such as autism and alexithymia. That is, our work indicates that the ability to form implicit personality judgements from faces in general appears to follow a unique automatic route in the cognitive system that warrants further investigation.

## Supporting information

S1 Table Correlational analysis for experiment 1a. (DOCX)

S2 Table Correlational analysis for experiment 1b. (DOCX)

S1 Appendix Additional analysis for Gender differences in IAT. (DOCX)

S2 Appendix Additional analysis for Accuracy scores in the IAT. (DOCX)
