# Supplementary material for: Do young and older adult populations perform equivalently across different automatic face-trait judgements? Evidence for differential impacts of ageing
Source: PLoS One. 2025 May 7;20(5):e0322165. doi: 10.1371/journal.pone.0322165 (PMC12057949; doi:10.1371/journal.pone.0322165)
Supplement: S1 Table — (DOCX) [file pone.0322165.s001.docx]

| Variable |  | Age | IAT | AQ | AQ1 | AQ2 | AQ3 | AQ4 | AQ5 | TAS20 | TAS1 | TAS2 | TAS3 |
| --- | --- | --- | --- | --- | --- | --- | --- | --- | --- | --- | --- | --- | --- |
| EIAT | *rs* | -0.165* | — |  |  |  |  |  |  |  |  |  |  |
|  | *p* | 0.027 | — |  |  |  |  |  |  |  |  |  |  |
| AQ | *rs* | 0.044 | -0.049 | — |  |  |  |  |  |  |  |  |  |
|  | *p* | 0.557 | 0.516 | — |  |  |  |  |  |  |  |  |  |
| AQ 1 | *rs* | -0.009 | -0.051 | 0.443*** | — |  |  |  |  |  |  |  |  |
|  | *p* | 0.903 | 0.493 | < .001 | — |  |  |  |  |  |  |  |  |
| AQ 2 | *rs* | -0.134 | 0.043 | 0.758*** | 0.082 | — |  |  |  |  |  |  |  |
|  | *p* | 0.072 | 0.570 | < .001 | 0.273 | — |  |  |  |  |  |  |  |
| AQ 3 | *rs* | 0.056 | -0.013 | 0.817*** | 0.142 | 0.623*** | — |  |  |  |  |  |  |
|  | *p* | 0.457 | 0.866 | < .001 | 0.057 | < .001 | — |  |  |  |  |  |  |
| AQ 4 | *rs* | 0.147* | -0.120 | 0.652*** | 0.214** | 0.371*** | 0.402*** | — |  |  |  |  |  |
|  | *p* | 0.049 | 0.110 | < .001 | 0.004 | < .001 | < .001 | — |  |  |  |  |  |
| AQ 5 | *rs* | 0.140 | -0.079 | 0.755*** | 0.030 | 0.536*** | 0.695*** | 0.406*** | — |  |  |  |  |
|  | *p* | 0.061 | 0.289 | < .001 | 0.685 | < .001 | < .001 | < .001 | — |  |  |  |  |
| TAS | *rs* | -0.150* | -0.062 | 0.520*** | 0.128 | 0.438*** | 0.491*** | 0.261*** | 0.442*** | — |  |  |  |
|  | *p* | 0.044 | 0.410 | < .001 | 0.087 | < .001 | < .001 | < .001 | < .001 | — |  |  |  |
| TAS 1 | *rs* | -0.192** | -0.108 | 0.405*** | 0.086 | 0.377*** | 0.353*** | 0.205** | 0.353*** | 0.843*** | — |  |  |
|  | *p* | 0.010 | 0.149 | < .001 | 0.251 | < .001 | < .001 | 0.006 | < .001 | < .001 | — |  |  |
| TAS 2 | *rs* | -0.013 | -0.063 | 0.272*** | -0.025 | 0.181* | 0.265*** | 0.209** | 0.310*** | 0.586*** | 0.300*** | — |  |
|  | *p* | 0.857 | 0.398 | < .001 | 0.741 | 0.015 | < .001 | 0.005 | < .001 | < .001 | < .001 | — |  |
| TAS 3 | *rs* | -0.115 | -0.031 | 0.496*** | 0.196** | 0.439*** | 0.470*** | 0.202** | 0.361*** | 0.851*** | 0.645*** | 0.235** | — |
|  | *p* | 0.123 | 0.678 | < .001 | 0.008 | < .001 | < .001 | 0.007 | < .001 | < .001 | < .001 | 0.001 | — |
| CFMT | *rs* | -0.245*** | 0.021 | -0.054 | 0.060 | 0.033 | -0.138 | -0.095 | -0.039 | -0.067 | -0.003 | -0.149* | -0.033 |
|  | *p* | < .001 | 0.785 | 0.475 | 0.426 | 0.657 | 0.065 | 0.207 | 0.604 | 0.372 | 0.972 | 0.046 | 0.658 |

**S1 Table. Correlational analysis for Experiment 1a**

Note. * *p* <.05, ** *p* < .01, *** *p* <.001.

Rs – Spearmans rho. EIAT – Extraversion Implicit Association task. AQ – Autism Quotient questionnaire. AQ subscales: AQ 1 – Attention to detail, AQ 2 – attention switching, AQ 3 – communication, AQ4 – Imagination, AQ 5 – Social Skills. TAS 20: Toronto alexithymia scale. TAS 20 subscales: TAS 1 – Difficulty describing feelings, TAS 2 – External thinking, TAS 3 – Identifying feelings. CFMT – Cambridge face memory task.
