# Supplementary material for: Do young and older adult populations perform equivalently across different automatic face-trait judgements? Evidence for differential impacts of ageing
Source: PLoS One. 2025 May 7;20(5):e0322165. doi: 10.1371/journal.pone.0322165 (PMC12057949; doi:10.1371/journal.pone.0322165)
Supplement: S2 Table — (DOCX) [file pone.0322165.s002.docx]

**S2 Table. Correlational Analysis for Experiment 1b.**

| Variable |  | Age | IAT | AQ | AQ1 | AQ2 | AQ3 | AQ4 | AQ5 | TAS20 | TAS1 | TAS2 | TAS3 | IPIP N |
| --- | --- | --- | --- | --- | --- | --- | --- | --- | --- | --- | --- | --- | --- | --- |
| NIAT | *rs* | 0.029 | — |  |  |  |  |  |  |  |  |  |  |  |
|  | *p* | 0.709 | — |  |  |  |  |  |  |  |  |  |  |  |
| AQ | *rs* | -0.197* | -0.080 | — |  |  |  |  |  |  |  |  |  |  |
|  | *p* | 0.010 | 0.300 | — |  |  |  |  |  |  |  |  |  |  |
| AQ 1 | *rs* | -0.079 | -0.081 | 0.517*** | — |  |  |  |  |  |  |  |  |  |
|  | *p* | 0.308 | 0.295 | < .001 | — | — |  |  |  |  |  |  |  |  |
| AQ 2 | *rs* | -0.255*** | -0.063 | 0.739*** | 0.222** | — |  |  |  |  |  |  |  |  |
|  | *p* | < .001 | 0.417 | < .001 | 0.004 | 0.483*** | — |  |  |  |  |  |  |  |
| AQ 3 | *rs* | -0.104 | -0.069 | 0.766*** | 0.209** | < .001 | — |  |  |  |  |  |  |  |
|  | *p* | 0.178 | 0.375 | < .001 | 0.006 | 0.193* | 0.314*** |  |  |  |  |  |  |  |
| AQ 4 | *rs* | -0.096 | -0.029 | 0.550*** | 0.128 | 0.012 | < .001 | — |  |  |  |  |  |  |
|  | *p* | 0.214 | 0.710 | < .001 | 0.097 | 0.539*** | 0.661*** | — |  |  |  |  |  |  |
| AQ 5 | *rs* | -0.070 | -0.064 | 0.797*** | 0.180* | < .001 | < .001 | 0.410*** | — |  |  |  |  |  |
|  | *p* | 0.363 | 0.405 | < .001 | 0.019 | 0.441*** | 0.313*** | < .001 | — |  |  |  |  |  |
| TAS | *rs* | -0.249** | 0.045 | 0.387*** | 0.154* | < .001 | < .001 | 0.085 | 0.258*** | — |  |  |  |  |
|  | *p* | 0.001 | 0.557 | < .001 | 0.045 | 0.446*** | 0.297*** | 0.272 | < .001 | — |  |  |  |  |
| TAS 1 | *rs* | -0.294*** | 0.005 | 0.413*** | 0.114 | < .001 | < .001 | 0.146 | 0.326*** | 0.844*** | — |  |  |  |
|  | *p* | < .001 | 0.950 | < .001 | 0.140 | 0.089 | 0.153* | 0.058 | < .001 | < .001 | — |  |  |  |
| TAS 2 | *rs* | -0.033 | 0.015 | 0.086 | -0.154* | 0.250 | 0.046 | 0.057 | 0.107 | 0.480*** | 0.222** | — |  |  |
|  | *p* | 0.667 | 0.850 | 0.263 | 0.045 | 0.416*** | 0.264*** | 0.460 | 0.165 | < .001 | 0.004 | — |  |  |
| TAS 3 | *rs* | -0.214** | 0.046 | 0.350*** | 0.264*** | < .001 | < .001 | 0.014 | 0.194* | 0.858*** | 0.667*** | 0.101 | — |  |
|  | *p* | 0.005 | 0.551 | < .001 | < .001 | 0.312*** | 0.256*** | 0.859 | 0.011 | < .001 | < .001 | 0.189 | — |  |
| IPIP N | *rs* | -0.235** | 0.042 | 0.276*** | 0.117 | < .001 | < .001 | 0.086 | 0.215** | 0.294*** | 0.238** | -0.049 | 0.413*** | — |
|  | *p* | 0.002 | 0.584 | < .001 | 0.130 | 0.156* | 0.031 | 0.264 | 0.005 | < .001 | 0.002 | 0.523 | < .001 | — |
| CFMT | *rs* | -0.154* | -0.104 | 0.105 | 0.141 | 0.042 | 0.683 | -0.136 | 0.079 | 0.012 | 0.037 | -0.143 | 0.061 | 0.089 |
|  | *p* | 0.045 | 0.177 | 0.171 | 0.066 |  |  | 0.076 | 0.304 | 0.880 | 0.628 | 0.063 | 0.426 | 0.250 |

Note. * *p* <.05, ** *p* < .01, *** *p* <.001.

Rs – Spearmans rho. NIAT – Implicit Association task. AQ – Autism Quotient questionnaire. AQ subscales: AQ 1 – Attention to detail, AQ 2 – attention switching, AQ 3 – communication, AQ4 – Imagination, AQ 5 – Social Skills. TAS 20: Toronto alexithymia scale. TAS 20 subscales: TAS 1 – Difficulty describing feelings, TAS 2 – External thinking, TAS 3 – Identifying feelings. CFMT – Cambridge face memory task. IPIP N – Self perception Neuroticism
