# Supplementary material for: Do young and older adult populations perform equivalently across different automatic face-trait judgements? Evidence for differential impacts of ageing
Source: PLoS One. 2025 May 7;20(5):e0322165. doi: 10.1371/journal.pone.0322165 (PMC12057949; doi:10.1371/journal.pone.0322165)
Supplement: S1 Appendix — (DOCX) [file pone.0322165.s003.docx]

**S3 Appendix.** **Additional analysis for Gender differences in IAT**

All data reported below were normally distributed. Independent sample t-tests were conducted on each of the 4 IATs (Young adults: Extraversion IAT, Neuroticism IAT; Older adults: Extraversion IAT, Neuroticism IAT) to determine whether performances in the IAT differed based on gender. All participants provided information on their gender. There were no significant differences in the performance of the IATs based on gender.

Young adults Extraversion face-trait judgements. There were no significant difference between Males (N = 50 , M = .107 , SD = .36 ) and Females (N = 68, M= .139, SD = .35), *t*(116) = .481, *p* = .63, Cohens *d* = .09.

Older adults Extraversion face-trait judgements. There were no significant difference between Males (N = 30, M= .02, SD= .47) and Females (N = 32, M= -.02, SD = .50), *t*(60) = -.30, *p* = .76, Cohens *d* = -.08

Young adults Neuroticism face-trait judgements. There were no significant difference between Males (N = 49, M= .145, SD = .42) and Females (N = 71, M= .178, SD = .38), *t*(118) = .46, *p* = .65, Cohens *d* =.09

Older adults Neuroticism face-trait judgements. There were no significant difference between Males (N = 19, M= .06, SD = .41) and Females (N = 31, M= .188, SD = .43), *t*(48) = 1.02, *p* = .31, Cohens *d* = .30
