# Supplementary material for: Do young and older adult populations perform equivalently across different automatic face-trait judgements? Evidence for differential impacts of ageing
Source: PLoS One. 2025 May 7;20(5):e0322165. doi: 10.1371/journal.pone.0322165 (PMC12057949; doi:10.1371/journal.pone.0322165)
Supplement: S2 Appendix — (DOCX) [file pone.0322165.s004.docx]

**S4 Appendix. Additional analysis for Accuracy scores in the IAT**

Accuracy scores were calculated by dividing the actual score by maximum possible score. Please see S4 Fig1 below for mean differences and standard error bar across all groups. Note that congruent denotes correct pairings where a highly extraverted face was paired with highly extraverted word (same for low extraversion; and for neuroticism), and incongruent denotes incorrect pairings where a highly extraverted face is paired with low extraversion words (vice versa for low extraverted face; and neuroticism). This is a standard IAT practice to showcase implicit bias. See Table S4 for Means and standard deviations by stimulus type (Image or word), and by block type (congruent, incongruent). Independent *t*-test showed no significant differences.

**S4 Fig 1. Mean differences in the IAT accuracy scores**


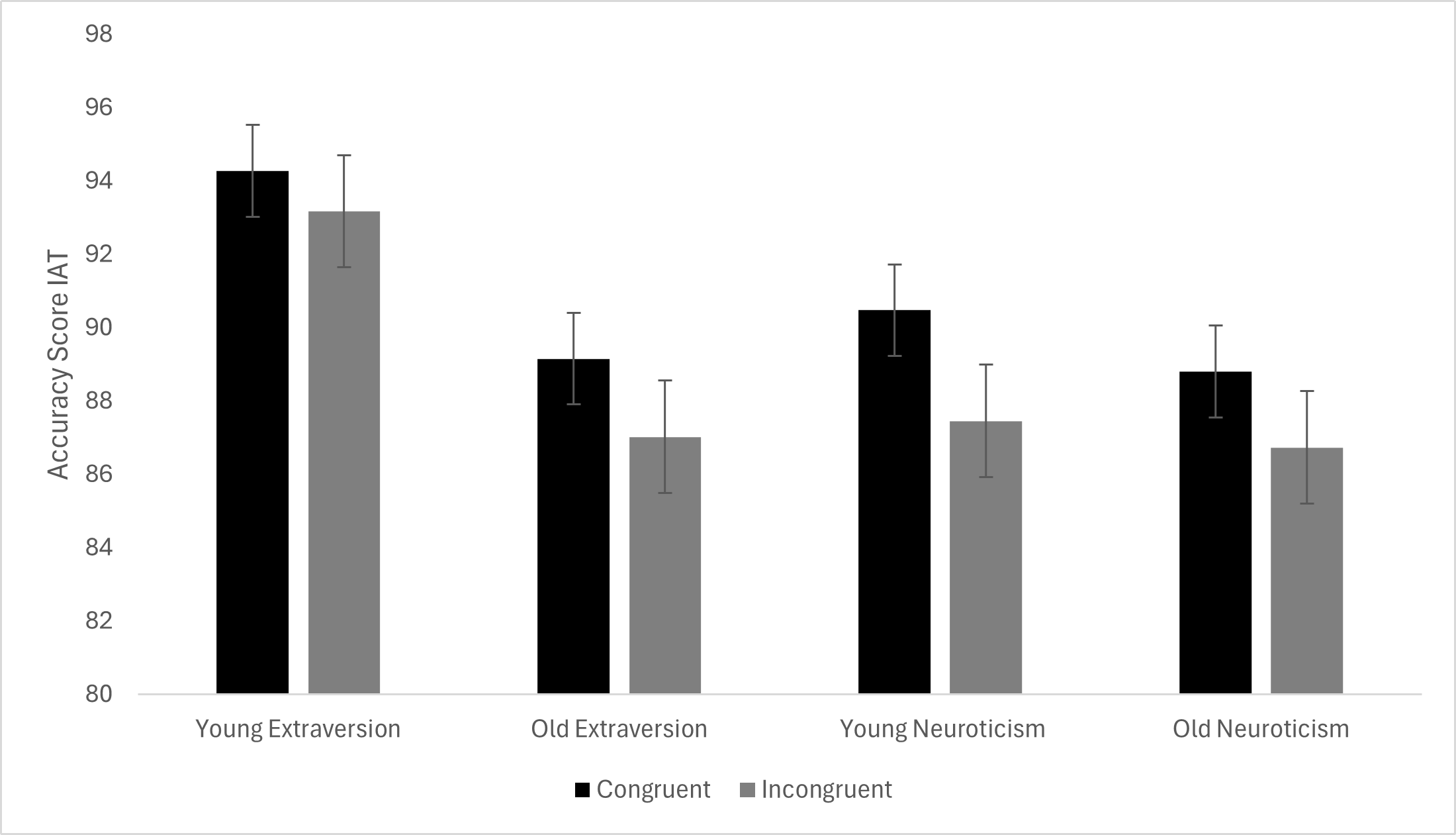


**Table S4. Accuracy scores by Stimulus type and Block type**

| Task | Congruent Word | Congruent Image | Incongruent Word | Incongruent Image |
| --- | --- | --- | --- | --- |
| Young Extraversion | *M=* 94.68 | *M=* 93.86 | *M=* 92.99 | *M=* 93.24 |
|  | *SD = 6.10* | *SD =* 7.26 | *SD = 8.13* | *SD =* 7.87 |
| Young Neuroticism | *M=* 90.69 | *M=* 90.25 | *M=* 86.67 | *M=* 88.23 |
|  | *SD =* 11.34 | *SD =* 11.86 | *SD =* 12.21 | *SD =* 14.53 |
| Old Extraversion | *M=* 94.64 | *M=* 83.67 | *M=* 95.08 | *M=* 78.95 |
|  | *SD =* 9.58 | *SD =* 16.77 | *SD =* 7.57 | *SD =* 20.36 |
| Old Neuroticism | *M=* 93.05 | *M=* 85.65 | *M=* 90.9 | *M=* 82.2 |
|  | *SD =* 12.04 | *SD =* 16.65 | *SD =* 12.73 | *SD =* 19.08 |
