# Supplementary material for: Do young and older adult populations perform equivalently across different automatic face-trait judgements? Evidence for differential impacts of ageing
Source: PLoS One. 2025 May 7;20(5):e0322165. doi: 10.1371/journal.pone.0322165 (PMC12057949; doi:10.1371/journal.pone.0322165)
Supplement: Attachment — Submitted filename: Response to Reviewers.docx [file pone.0322165.s006.docx]

PONE-D-24-06206

Dear Reviewers and Editorial Team,

Thank you for taking the time and effort to provide extremely valuable feedback on the article titled ‘Do young and older adult populations perform equivalently across different automatic face-trait judgements? Evidence for differential impacts of ageing.’ I have included my responses to each of the questions raised below.

**Editorial Team - Journal Requirements**

1.  Please ensure that your manuscript meets PLOS ONE's style requirements, including those for file naming. The PLOS ONE style templates can be found at

<https://journals.plos.org/plosone/s/file?id=wjVg/PLOSOne_formatting_sample_main_body.pdf>  and <https://journals.plos.org/plosone/s/file?id=ba62/PLOSOne_formatting_sample_title_authors_affiliations.pdf>

- The changes are reflected throughout the manuscript. Please see the highlighted content with track changes. Amendments to figure formats have been addressed using PACE, converted to tiff files and uploaded to the manuscript.

2. When completing the data availability statement of the submission form, you indicated that you will make your data available on acceptance.

We strongly recommend all authors decide on a data sharing plan before acceptance, as the process can be lengthy and hold up publication timelines. Please note that, though access restrictions are acceptable now, your entire data will need to be made freely accessible if your manuscript is accepted for publication. This policy applies to all data except where public deposition would breach compliance with the protocol approved by your research ethics board. If you are unable to adhere to our open data policy, please kindly revise your statement to explain your reasoning and we will seek the editor's input on an exemption. Please be assured that, once you have provided your new statement, the assessment of your exemption will not hold up the peer review process.

- This is an anonymised data set that is already made available on the Open Science Framework <https://osf.io/4nvgw/>. On publication, this will include public access.

3. We note that Figure 1 includes an image of a participant in the study.

As per the PLOS ONE policy (http://journals.plos.org/plosone/s/submission-guidelines#loc-human-subjects-research) on papers that include identifying, or potentially identifying, information, the individual(s) or parent(s)/guardian(s) must be informed of the terms of the PLOS open-access (CC-BY) license and provide specific permission for publication of these details under the terms of this license. Please download the Consent Form for Publication in a PLOS Journal (http://journals.plos.org/plosone/s/file?id=8ce6/plos-consent-form-english.pdf). The signed consent form should not be submitted with the manuscript, but should be securely filed in the individual's case notes. Please amend the methods section and ethics statement of the manuscript to explicitly state that the patient/participant has provided consent for publication: “The individual in this manuscript has given written informed consent (as outlined in PLOS consent form) to publish these case details”.

If you are unable to obtain consent from the subject of the photograph, you will need to remove the figure and any other textual identifying information or case descriptions for this individual.

- This is a composite (average faces) image that was digitally created by the authors for the purpose of this study. Please note that this is NOT an image of a participant in this study, and does not correspond to any participant. As such, this is not identifiable information. Although there might be some similarities, the average image included does not involve a single identifiable individual. Hence human consent is not necessary here. All participants in the study provided written informed consent for the same. One of the authors from the original study is also an author in this paper.

4. We note that Figure 2 in your submission contain copyrighted images. All PLOS content is published under the Creative Commons Attribution License (CC BY 4.0), which means that the manuscript, images, and Supporting Information files will be freely available online, and any third party is permitted to access, download, copy, distribute, and use these materials in any way, even commercially, with proper attribution. For more information, see our copyright guidelines: <http://journals.plos.org/plosone/s/licenses-and-copyright>.

We require you to either (1) present written permission from the copyright holder to publish these figures specifically under the CC BY 4.0 license, or (2) remove the figures from your submission:

a. You may seek permission from the original copyright holder of Figure 2 to publish the content specifically under the CC BY 4.0 license.

We recommend that you contact the original copyright holder with the Content Permission Form (http://journals.plos.org/plosone/s/file?id=7c09/content-permission-form.pdf) and the following text:

“I request permission for the open-access journal PLOS ONE to publish XXX under the Creative Commons Attribution License (CCAL) CC BY 4.0 (http://creativecommons.org/licenses/by/4.0/). Please be aware that this license allows unrestricted use and distribution, even commercially, by third parties. Please reply and provide explicit written permission to publish XXX under a CC BY license and complete the attached form.”

Please upload the completed Content Permission Form or other proof of granted permissions as an "Other" file with your submission.

In the figure caption of the copyrighted figure, please include the following text: “Reprinted from [ref] under a CC BY license, with permission from [name of publisher], original copyright [original copyright year].”

b. If you are unable to obtain permission from the original copyright holder to publish these figures under the CC BY 4.0 license or if the copyright holder’s requirements are incompatible with the CC BY 4.0 license, please either i) remove the figure or ii) supply a replacement figure that complies with the CC BY 4.0 license. Please check copyright information on all replacement figures and update the figure caption with source information. If applicable, please specify in the figure caption text when a figure is similar but not identical to the original image and is therefore for illustrative purposes only.

- the images were obtained from KDEF database. The current study employed the design by Palermo et al. (2013). This has now been amended in the manuscript. Please see note to author and publisher from the database website included below (<https://kdef.se/faq/using-and-publishing-kdef-and-akdef>).

“**PUBLISHING KDEF or AKDEF STIMULI**

To the author:

1. If a KDEF or AKDEF stimulus is included in a figure in your manuscript; please include information about the KDEF or AKDEF image id in the figure text (F01HAS or similar).
2. If possible, the manuscript should also include a list of the KDEF or AKDEF ids of all the stimuli used in the experiment, under Methods>Stimuli or similar

To the publisher:

1. Researchers may always include sample images from KDEF or AKDEF in his/her manuscript when said manuscript is a doctoral thesis OR is a manuscript submitted to a scientific journal. A publisher may regard this mail as a written consent for such publication, or contact me (contact info below) directly if needed.
   1. For the KDEF stimuli, such a journal is typically PLOS ONE, EMOTION, NEUROPSYCHOLOGIA, COGNITION & EMOTION, SOCIAL COGNITIVE & AFFECTIVE NEUROSCIENCE, BIOLOGICAL PSYCHOLOGY, NEUROIMAGE, FRONTIERS IN PSYCHOLOGY, JOURNAL OF NEUROSCIENCE or PSYCHONEUROENDOCRINOLOGY. Or similar.
2. For already published articles using and referencing to KDEF, see [*here*](https://scholar.google.se/citations?view_op=view_citation&hl=sv&user=Z2xS-FMAAAAJ&citation_for_view=Z2xS-FMAAAAJ:u5HHmVD_uO8C).
3. For other type of publications, please contact me – contact info below.
4. Apart from the above publication purposes, the KDEF and AKDEF stimuli may NOT be redistributed or shared without written consent from the copyright holder (Karolinska Institutet, Psychology section; contact info below).”

**Response to Reviewer 1**

1.The introduction is quite lengthy. It is recommended to condense the introduction to improve focus and readability.

- The authors have tried to make the manuscript as concise as possible. Changes are reflected in the revised manuscript.

2. The alignment in the results section could be more streamlined to improve clarity and flow of information.

- Changes are reflected in the manuscript by including sub-headings and additional notes on the statistical measures.

3. Could the author provide an explanation of how the automatic trait judgments observed in this study might generalize to different cultural contexts, where facial expressions and personality traits may be perceived differently?

- This study was controlled for other-ethnicity. Only Caucasian sample was included in this study. The authors have also conducted a follow up study using the same IAT paradigm in other-ethnicity samples (currently under prep), there was no IAT effects here, thus showing that culture does play a role, but this needs to also be tested more rigorously by testing Caucasian group with the other ethnicity faces. Also see paper Meisser and Bringham, (2001) <https://psycnet.apa.org/buy/2001-14540-001>, and this shows other-ethnicity effects with other concepts of face recognition, but not personality.

4. Are there cultural differences in interpreting the composite facial stimuli used for implicit personality trait judgments? How might this affect the findings?

- As stated in the response to point 3 above, given other-ethnicity effects can play a role (identified in follow up study), the current study has controlled for this factor by only including Caucasian samples. Thus, it does not affect the current findings as it stands. As an Indian researcher, I have rigorously thought through these concepts with lived experience in other cultures. These points noted by the reviewing team are highly insightful.

5. In older adults, is there a correlation between autism traits and the ability to recognize facial cues related to neuroticism and extraversion?

- There were no correlations reported for IAT and AQ in older adults in both extraversion and neuroticism experiments. Only significant correlations are reported in the results section. All correlational matrices are included in the supplementary materials.

6. How does the inclusion of error latency in the IAT scoring algorithm (as applied in this study) affect the overall accuracy of D-scores, especially considering possible cognitive differences between age groups or individuals with traits like high alexithymia?

- There is extensive analysis of the IAT D algorithm that suggests the improved scoring algorithm introduced by Greenwald et al. (2003) is the most effective approach. Greenwald and colleagues developed the D-score as a more reliable measure of implicit attitudes by correcting for individual differences in response times and error rates. Their scoring method adjusts for error latencies by replacing incorrect responses with the average response time from the block plus 600 milliseconds to account for errors. This adjustment ensures that response times (RTs) are not unduly influenced by mistakes made by participants. The goal was to minimize the impact of random errors and to more accurately measure implicit associations. In our study, we deviated from the original method by utilizing the time taken until participants provided correct responses, rather than simply adding 600 milliseconds to the error latencies. This approach better reflects the participants' response patterns and aligns with the concept employed by Jones et al. (2019). Additionally, we conducted a simple analysis to determine whether different age groups made more errors compared to others by conducting t-tests on accuracy scores; however, we found no significant differences (This has also been included in supplementary materials S4). Overall, the current approach of using the improved scoring algorithm, as described, and compared against other possible scoring mechanisms proposed by Greenwald, represents the most accurate way to analyse the IAT.

7. Are there additional modifications or refinements to the IAT scoring algorithm that could improve its validity in measuring implicit personality traits, particularly in diverse populations such as older adults or individuals with autism traits?

- The error correction applied in the scoring algorithm, similar to the approach by Jones et al. (2019), already addresses one way to enhance the scoring system. Work by Gonsalkorale et al. (2009) suggests people who are older might actually be better measured by the IAT (<https://www.sciencedirect.com/science/article/abs/pii/S0022103108002370>) . While a more refined scoring algorithm might be possible, it would require extensive testing. Additionally, some form of modeling approach could be considered; however, we strongly believe it is best to adhere to the traditional methods employed in the established IAT algorithms, as deviating from these practices may not be advisable.

**Response to Reviewer 2**

1. The description of the IAT task is not easy for people unfamiliar with the test to understand. Image examples are given, but word examples were only given in the text but not the figures. A couple examples of the blocks with corresponding image/word pairs would be helpful.

- This has been amended in the manuscript by including IAT block design as an example. Please see page 12, line 277, figure 2.

2. No example images for the Cambridge Face Memory task (CFMT) were shown. Were the composites all from female Caucasian, as in the IAT task? Or were they more diverse? Can this affect the interpretation on the results regarding the effect of face memory on implicit personality trait judgement?

- This is an excellent point. Given the CFMT is a well-established measure, example stimuli were not included in our study. Additionally, we don’t have copyright agreement for the image. Please see [CFMT](https://www.sciencedirect.com/science/article/pii/S0028393205002496?casa_token=6FTs_bHj8SYAAAAA:Vdwy03-WWC6qpNQBwMlsJkodUmnbeuBX7y7mglAxoL3PQNnxB1aMhjfB2XbCI8Ztebb8D7ij) for original paper if you would like to view the images used in the study. The stimuli used in CFMT includes Caucasian young male faces. There is mixed evidence for gender differences in face memory performances (e.g. Scherf et al., 2017; Bobak et al., 2019). However, the effects are very minimal and not well established. As such, this should not affect the results of the current study. Female faces CFMT is not as robust as male CFMT. This still requires a lot of test re-test reliability. However, we will certainly consider using this in the future.

  3. Regarding statistical tests, for either young or older adults in the IAT extraversion test, one-sample t-test was used for the D scores (Line 354), but when comparing between young and old, and Mann-Whitney (Line 368) was used. Were these two groups normally distributed or not?
- The data was normally distributed. An independent t-test results are included along with effect size estimates. Changes are reflected in the manuscript.

  4. Line 358-360: “Young adults were faster and more accurate on trials where highly extraverted faces were paired with highly extraverted words, and on trials where lower trait faces were paired with lower trait words.” Need to clarify which part of the data support this claim (e.g. a significantly positive D score, etc). Related, an explanation on the interpretation of the D score would be helpful, such as what a non-zero score mean, in addition to defining the value mathematically.
- When IAT D scores are approximately 0, it denotes that there is not much difference in response latency between congruent (correct pairings) and incongruent (incorrect) conditions. Additional line as per suggestion, has been included on the amended manuscript on what positive D score means.

  5. Was there a sex difference in any of the results, especially when only female composites were used?
- Although this wasn’t the main focus of the study, there were no gender differences across the IATs. This was tested using independent t-tests, yielding no significant differences in gender. This has now been included in the supplementary material Appendix S3.

  6. For the neuroticism group, Line 205 indicates there were 120 young adults but in Table 5 the number is 118.
- Error amended. There are 120 young adults in the neuroticism study.
