# Supplementary material for: Do young and older adult populations perform equivalently across different automatic face-trait judgements? Evidence for differential impacts of ageing
Source: PLoS One. 2025 May 7;20(5):e0322165. doi: 10.1371/journal.pone.0322165 (PMC12057949; doi:10.1371/journal.pone.0322165)
Supplement: Peer review history

# Author comment: Author response to Decision Letter 0

**DOI:** 10.1371/journal.pone.0322165.r001

26 Feb 2024

# Peer review: Decision Letter 0

**DOI:** 10.1371/journal.pone.0322165.r002

**Authors:** Hosam Al-Samarraie

14 Jan 2025

PONE-D-24-06206Do young and older adult populations perform equivalently across different automatic face-trait judgements? Evidence for differential impacts of ageing.PLOS ONE

Dear Dr. Kannan,

Thank you for submitting your manuscript to PLOS ONE. After careful consideration, we feel that it has merit but does not fully meet PLOS ONE’s publication criteria as it currently stands. Therefore, we invite you to submit a revised version of the manuscript that addresses the points raised during the review process.

Please submit your revised manuscript by Feb 28 2025 11:59PM. If you will need more time than this to complete your revisions, please reply to this message or contact the journal office at plosone@plos.org . When you're ready to submit your revision, log on to https://www.editorialmanager.com/pone/ and select the 'Submissions Needing Revision' folder to locate your manuscript file.

Please include the following items when submitting your revised manuscript:

- A rebuttal letter that responds to each point raised by the academic editor and reviewer(s). You should upload this letter as a separate file labeled 'Response to Reviewers'.
- A marked-up copy of your manuscript that highlights changes made to the original version. You should upload this as a separate file labeled 'Revised Manuscript with Track Changes'.
- An unmarked version of your revised paper without tracked changes. You should upload this as a separate file labeled 'Manuscript'.

If you would like to make changes to your financial disclosure, please include your updated statement in your cover letter. Guidelines for resubmitting your figure files are available below the reviewer comments at the end of this letter.

If applicable, we recommend that you deposit your laboratory protocols in protocols.io to enhance the reproducibility of your results. Protocols.io assigns your protocol its own identifier (DOI) so that it can be cited independently in the future. For instructions see: https://journals.plos.org/plosone/s/submission-guidelines#loc-laboratory-protocols . Additionally, PLOS ONE offers an option for publishing peer-reviewed Lab Protocol articles, which describe protocols hosted on protocols.io. Read more information on sharing protocols at https://plos.org/protocols?utm_medium=editorial-email&utm_source=authorletters&utm_campaign=protocols .

We look forward to receiving your revised manuscript.

Kind regards,

Hosam Al-Samarraie

Academic Editor

PLOS ONE

Journal requirements:

When submitting your revision, we need you to address these additional requirements.

1.  Please ensure that your manuscript meets PLOS ONE's style requirements, including those for file naming. The PLOS ONE style templates can be found at

https://journals.plos.org/plosone/s/file?id=wjVg/PLOSOne_formatting_sample_main_body.pdf   and

https://journals.plos.org/plosone/s/file?id=ba62/PLOSOne_formatting_sample_title_authors_affiliations.pdf

2. When completing the data availability statement of the submission form, you indicated that you will make your data available on acceptance. We strongly recommend all authors decide on a data sharing plan before acceptance, as the process can be lengthy and hold up publication timelines. Please note that, though access restrictions are acceptable now, your entire data will need to be made freely accessible if your manuscript is accepted for publication. This policy applies to all data except where public deposition would breach compliance with the protocol approved by your research ethics board. If you are unable to adhere to our open data policy, please kindly revise your statement to explain your reasoning and we will seek the editor's input on an exemption. Please be assured that, once you have provided your new statement, the assessment of your exemption will not hold up the peer review process.

3. We note that Figure 1 includes an image of a participant in the study.

As per the PLOS ONE policy (http://journals.plos.org/plosone/s/submission-guidelines#loc-human-subjects-research) on papers that include identifying, or potentially identifying, information, the individual(s) or parent(s)/guardian(s) must be informed of the terms of the PLOS open-access (CC-BY) license and provide specific permission for publication of these details under the terms of this license. Please download the Consent Form for Publication in a PLOS Journal (http://journals.plos.org/plosone/s/file?id=8ce6/plos-consent-form-english.pdf). The signed consent form should not be submitted with the manuscript, but should be securely filed in the individual's case notes. Please amend the methods section and ethics statement of the manuscript to explicitly state that the patient/participant has provided consent for publication: “The individual in this manuscript has given written informed consent (as outlined in PLOS consent form) to publish these case details”.

If you are unable to obtain consent from the subject of the photograph, you will need to remove the figure and any other textual identifying information or case descriptions for this individual.

4. We note that Figure 2 in your submission contain copyrighted images. All PLOS content is published under the Creative Commons Attribution License (CC BY 4.0), which means that the manuscript, images, and Supporting Information files will be freely available online, and any third party is permitted to access, download, copy, distribute, and use these materials in any way, even commercially, with proper attribution. For more information, see our copyright guidelines: http://journals.plos.org/plosone/s/licenses-and-copyright.

We require you to either (1) present written permission from the copyright holder to publish these figures specifically under the CC BY 4.0 license, or (2) remove the figures from your submission:

a. You may seek permission from the original copyright holder of Figure 2 to publish the content specifically under the CC BY 4.0 license.

We recommend that you contact the original copyright holder with the Content Permission Form (http://journals.plos.org/plosone/s/file?id=7c09/content-permission-form.pdf) and the following text:

“I request permission for the open-access journal PLOS ONE to publish XXX under the Creative Commons Attribution License (CCAL) CC BY 4.0 (http://creativecommons.org/licenses/by/4.0/). Please be aware that this license allows unrestricted use and distribution, even commercially, by third parties. Please reply and provide explicit written permission to publish XXX under a CC BY license and complete the attached form.”

Please upload the completed Content Permission Form or other proof of granted permissions as an "Other" file with your submission.

In the figure caption of the copyrighted figure, please include the following text: “Reprinted from [ref] under a CC BY license, with permission from [name of publisher], original copyright [original copyright year].”

b. If you are unable to obtain permission from the original copyright holder to publish these figures under the CC BY 4.0 license or if the copyright holder’s requirements are incompatible with the CC BY 4.0 license, please either i) remove the figure or ii) supply a replacement figure that complies with the CC BY 4.0 license. Please check copyright information on all replacement figures and update the figure caption with source information. If applicable, please specify in the figure caption text when a figure is similar but not identical to the original image and is therefore for illustrative purposes only.

[Note: HTML markup is below. Please do not edit.]

Reviewers' comments:

Reviewer's Responses to Questions

**Comments to the Author**

1. Is the manuscript technically sound, and do the data support the conclusions?

The manuscript must describe a technically sound piece of scientific research with data that supports the conclusions. Experiments must have been conducted rigorously, with appropriate controls, replication, and sample sizes. The conclusions must be drawn appropriately based on the data presented. 

Reviewer #1: Partly

Reviewer #2: Yes

**********

2. Has the statistical analysis been performed appropriately and rigorously? 

Reviewer #1: I Don't Know

Reviewer #2: No

**********

3. Have the authors made all data underlying the findings in their manuscript fully available?

The PLOS Data policy requires authors to make all data underlying the findings described in their manuscript fully available without restriction, with rare exception (please refer to the Data Availability Statement in the manuscript PDF file). The data should be provided as part of the manuscript or its supporting information, or deposited to a public repository. For example, in addition to summary statistics, the data points behind means, medians and variance measures should be available. If there are restrictions on publicly sharing data—e.g. participant privacy or use of data from a third party—those must be specified.

Reviewer #1: Yes

Reviewer #2: No

**********

4. Is the manuscript presented in an intelligible fashion and written in standard English?

PLOS ONE does not copyedit accepted manuscripts, so the language in submitted articles must be clear, correct, and unambiguous. Any typographical or grammatical errors should be corrected at revision, so please note any specific errors here.

Reviewer #1: No

Reviewer #2: Yes

**********

5. Review Comments to the Author

Please use the space provided to explain your answers to the questions above. You may also include additional comments for the author, including concerns about dual publication, research ethics, or publication ethics. (Please upload your review as an attachment if it exceeds 20,000 characters)

Reviewer #1: In the current study, the author investigated whether implicit personality trait judgments can be made accurately across different age groups, specifically comparing younger and older adults. The research focused on two traits—extraversion (positive) and neuroticism (negative)—using faces as stimuli. The findings revealed that younger participants were accurate in making automatic judgments for both traits, whereas older adults showed similar accuracy for neuroticism judgments but exhibited impairments in extraversion judgments.

Comments to the Author:

1. The introduction is quite lengthy. It is recommended to condense the introduction to improve focus and readability.

2. The alignment in the results section could be more streamlined to improve clarity and flow of information.

3. Could the author provide an explanation of how the automatic trait judgments observed in this study might generalize to different cultural contexts, where facial expressions and personality traits may be perceived differently?

4. Are there cultural differences in interpreting the composite facial stimuli used for implicit personality trait judgments? How might this affect the findings?

5. In older adults, is there a correlation between autism traits and the ability to recognize facial cues related to neuroticism and extraversion?

6. How does the inclusion of error latency in the IAT scoring algorithm (as applied in this study) affect the overall accuracy of D-scores, especially considering possible cognitive differences between age groups or individuals with traits like high alexithymia?

7. Are there additional modifications or refinements to the IAT scoring algorithm that could improve its validity in measuring implicit personality traits, particularly in diverse populations such as older adults or individuals with autism traits?

Reviewer #2: In the manuscript titled “Do young and older adult populations perform equivalently across different automatic face-trait judgements? Evidence for differential impacts of ageing”, the authors used the Implicit Association Task (IAT) to investigate age-related changes in implicit personality trait judgement. Using a battery of tests, the authors also examined the relationship between implicit personality trait judgement and factors such as facial memory, emotion perception, autism traits, and alexithymia traits. The results showed that young adults can form accurate implicit associations for both extraversion and neuroticism traits, while older adults showed deficits in the former but not the latter, indicating differential effect of ageing on different aspect of implicit trait judgement. The manuscript is well written, and the results are interesting and novel. However, I do have the following comments:

1. The description of the IAT task is not easy for people unfamiliar with the test to understand. Image examples are given, but word examples were only given in the text but not the figures. A couple examples of the blocks with corresponding image/word pairs would be helpful.

2. No example images for the Cambridge Face Memory task (CFMT) were shown. Were the composites all from female Caucasian, as in the IAT task? Or were they more diverse? Can this affect the interpretation on the results regarding the effect of face memory on implicit personality trait judgement?

3. Regarding statistical tests, for either young or older adults in the IAT extraversion test, one-sample t-test was used for the D scores (Line 354), but when comparing between young and old, and Mann-Whitney (Line 368) was used. Were these two groups normally distributed or not?

4. Line 358-360: “Young adults were faster and more accurate on trials where highly extraverted faces were paired with highly extraverted words, and on trials where lower trait faces were paired with lower trait words.” Need to clarify which part of the data support this claim (e.g. a significantly positive D score, etc). Related, an explanation on the interpretation of the D score would be helpful, such as what a non-zero score mean, in addition to defining the value mathematically.

5. Was there a sex difference in any of the results, especially when only female composites were used?

6. For the neuroticism group, Line 205 indicates there were 120 young adults but in Table 5 the number is 118.

**********

6. PLOS authors have the option to publish the peer review history of their article (what does this mean? ). If published, this will include your full peer review and any attached files.

If you choose “no”, your identity will remain anonymous but your review may still be made public.

**Do you want your identity to be public for this peer review?** For information about this choice, including consent withdrawal, please see our Privacy Policy .

Reviewer #1: No

Reviewer #2: No

**********

[NOTE: If reviewer comments were submitted as an attachment file, they will be attached to this email and accessible via the submission site. Please log into your account, locate the manuscript record, and check for the action link "View Attachments". If this link does not appear, there are no attachment files.]

While revising your submission, please upload your figure files to the Preflight Analysis and Conversion Engine (PACE) digital diagnostic tool, https://pacev2.apexcovantage.com/ . PACE helps ensure that figures meet PLOS requirements. To use PACE, you must first register as a user. Registration is free. Then, login and navigate to the UPLOAD tab, where you will find detailed instructions on how to use the tool. If you encounter any issues or have any questions when using PACE, please email PLOS at figures@plos.org . Please note that Supporting Information files do not need this step.

# Author comment: Author response to Decision Letter 1

**DOI:** 10.1371/journal.pone.0322165.r003

28 Feb 2025

Dear Review team,

Thank you very much for taking the time to provide immensely valuable feedback on our paper. All suggested changes have been addressed in the manuscript. I am including my response below to each of your questions.

Editorial Team - Journal Requirements

1. Please ensure that your manuscript meets PLOS ONE's style requirements, including those for file naming. The PLOS ONE style templates can be found at

https://journals.plos.org/plosone/s/file?id=wjVg/PLOSOne_formatting_sample_main_body.pdf and https://journals.plos.org/plosone/s/file?id=ba62/PLOSOne_formatting_sample_title_authors_affiliations.pdf

- The changes are reflected throughout the manuscript. Please see the highlighted content with track changes. Amendments to figure formats have been addressed using PACE, converted to tiff files and uploaded to the manuscript.

2. When completing the data availability statement of the submission form, you indicated that you will make your data available on acceptance.

We strongly recommend all authors decide on a data sharing plan before acceptance, as the process can be lengthy and hold up publication timelines. Please note that, though access restrictions are acceptable now, your entire data will need to be made freely accessible if your manuscript is accepted for publication. This policy applies to all data except where public deposition would breach compliance with the protocol approved by your research ethics board. If you are unable to adhere to our open data policy, please kindly revise your statement to explain your reasoning and we will seek the editor's input on an exemption. Please be assured that, once you have provided your new statement, the assessment of your exemption will not hold up the peer review process.

- This is an anonymised data set that is already made available on the Open Science Framework https://osf.io/4nvgw/. On publication, this will include public access.

3. We note that Figure 1 includes an image of a participant in the study.

As per the PLOS ONE policy (http://journals.plos.org/plosone/s/submission-guidelines#loc-human-subjects-research) on papers that include identifying, or potentially identifying, information, the individual(s) or parent(s)/guardian(s) must be informed of the terms of the PLOS open-access (CC-BY) license and provide specific permission for publication of these details under the terms of this license. Please download the Consent Form for Publication in a PLOS Journal (http://journals.plos.org/plosone/s/file?id=8ce6/plos-consent-form-english.pdf). The signed consent form should not be submitted with the manuscript, but should be securely filed in the individual's case notes. Please amend the methods section and ethics statement of the manuscript to explicitly state that the patient/participant has provided consent for publication: “The individual in this manuscript has given written informed consent (as outlined in PLOS consent form) to publish these case details”.

If you are unable to obtain consent from the subject of the photograph, you will need to remove the figure and any other textual identifying information or case descriptions for this individual.

- This is a composite (average faces) image that was digitally created by the authors for the purpose of this study. Please note that this is NOT an image of a participant in this study, and does not correspond to any participant. As such, this is not identifiable information. Although there might be some similarities, the average image included does not involve a single identifiable individual. Hence human consent is not necessary here. All participants in the study provided written informed consent for the same. One of the authors from the original study is also an author in this paper.

4. We note that Figure 2 in your submission contain copyrighted images. All PLOS content is published under the Creative Commons Attribution License (CC BY 4.0), which means that the manuscript, images, and Supporting Information files will be freely available online, and any third party is permitted to access, download, copy, distribute, and use these materials in any way, even commercially, with proper attribution. For more information, see our copyright guidelines: http://journals.plos.org/plosone/s/licenses-and-copyright.

We require you to either (1) present written permission from the copyright holder to publish these figures specifically under the CC BY 4.0 license, or (2) remove the figures from your submission:

a. You may seek permission from the original copyright holder of Figure 2 to publish the content specifically under the CC BY 4.0 license.

We recommend that you contact the original copyright holder with the Content Permission Form (http://journals.plos.org/plosone/s/file?id=7c09/content-permission-form.pdf) and the following text:

“I request permission for the open-access journal PLOS ONE to publish XXX under the Creative Commons Attribution License (CCAL) CC BY 4.0 (http://creativecommons.org/licenses/by/4.0/). Please be aware that this license allows unrestricted use and distribution, even commercially, by third parties. Please reply and provide explicit written permission to publish XXX under a CC BY license and complete the attached form.”

Please upload the completed Content Permission Form or other proof of granted permissions as an "Other" file with your submission.

In the figure caption of the copyrighted figure, please include the following text: “Reprinted from [ref] under a CC BY license, with permission from [name of publisher], original copyright [original copyright year].”

b. If you are unable to obtain permission from the original copyright holder to publish these figures under the CC BY 4.0 license or if the copyright holder’s requirements are incompatible with the CC BY 4.0 license, please either i) remove the figure or ii) supply a replacement figure that complies with the CC BY 4.0 license. Please check copyright information on all replacement figures and update the figure caption with source information. If applicable, please specify in the figure caption text when a figure is similar but not identical to the original image and is therefore for illustrative purposes only.

- The images were obtained from KDEF database. The current study employed the design by Palermo et al. (2013). This has now been amended in the manuscript. Please see note to author and publisher from the database website included below (https://kdef.se/faq/using-and-publishing-kdef-and-akdef).

“PUBLISHING KDEF or AKDEF STIMULI

To the author:

1. If a KDEF or AKDEF stimulus is included in a figure in your manuscript; please include information about the KDEF or AKDEF image id in the figure text (F01HAS or similar).

2. If possible, the manuscript should also include a list of the KDEF or AKDEF ids of all the stimuli used in the experiment, under Methods>Stimuli or similar

To the publisher:

1. Researchers may always include sample images from KDEF or AKDEF in his/her manuscript when said manuscript is a doctoral thesis OR is a manuscript submitted to a scientific journal. A publisher may regard this mail as a written consent for such publication, or contact me (contact info below) directly if needed.

1. For the KDEF stimuli, such a journal is typically PLOS ONE, EMOTION, NEUROPSYCHOLOGIA, COGNITION & EMOTION, SOCIAL COGNITIVE & AFFECTIVE NEUROSCIENCE, BIOLOGICAL PSYCHOLOGY, NEUROIMAGE, FRONTIERS IN PSYCHOLOGY, JOURNAL OF NEUROSCIENCE or PSYCHONEUROENDOCRINOLOGY. Or similar.

2. For already published articles using and referencing to KDEF, see here.

3. For other type of publications, please contact me – contact info below.

4. Apart from the above publication purposes, the KDEF and AKDEF stimuli may NOT be redistributed or shared without written consent from the copyright holder (Karolinska Institutet, Psychology section; contact info below).”

Response to Reviewer 1

1.The introduction is quite lengthy. It is recommended to condense the introduction to improve focus and readability.

- The authors have tried to make the manuscript as concise as possible. Changes are reflected in the revised manuscript.

2. The alignment in the results section could be more streamlined to improve clarity and flow of information.

- Changes are reflected in the manuscript by including sub-headings and additional notes on the statistical measures.

3. Could the author provide an explanation of how the automatic trait judgments observed in this study might generalize to different cultural contexts, where facial expressions and personality traits may be perceived differently?

- This study was controlled for other-ethnicity. Only Caucasian sample was included in this study. The authors have also conducted a follow up study using the same IAT paradigm in other-ethnicity samples (currently under prep), there was no IAT effects here, thus showing that culture does play a role, but this needs to also be tested more rigorously by testing Caucasian group with the other ethnicity faces. Also see paper Meisser and Bringham, (2001) https://psycnet.apa.org/buy/2001-14540-001, and this shows other-ethnicity effects with other concepts of face recognition, but not personality.

4. Are there cultural differences in interpreting the composite facial stimuli used for implicit personality trait judgments? How might this affect the findings?

- As stated in the response to point 3 above, given other-ethnicity effects can play a role (identified in follow up study), the current study has controlled for this factor by only including Caucasian samples. Thus, it does not affect the current findings as it stands. As an Indian researcher, I have rigorously thought through these concepts with lived experience in other cultures. These points noted by the reviewing team are highly insightful.

5. In older adults, is there a correlation between autism traits and the ability to recognize facial cues related to neuroticism and extraversion?

- There were no correlations reported for IAT and AQ in older adults in both extraversion and neuroticism experiments. Only significant correlations are reported in the results section. All correlational matrices are included in the supplementary materials.

6. How does the inclusion of error latency in the IAT scoring algorithm (as applied in this study) affect the overall accuracy of D-scores, especially considering possible cognitive differences between age groups or individuals with traits like high alexithymia?

- There is extensive analysis of the IAT D algorithm that suggests the improved scoring algorithm introduced by Greenwald et al. (2003) is the most effective approach. Greenwald and colleagues developed the D-score as a more reliable measure of implicit attitudes by correcting for individual differences in response times and error rates. Their scoring method adjusts for error latencies by replacing incorrect responses with the average response time from the block plus 600 milliseconds to account for errors. This adjustment ensures that response times (RTs) are not unduly influenced by mistakes made by participants. The goal was to minimize the impact of random errors and to more accurately measure implicit associations. In our study, we deviated from the original method by utilizing the time taken until participants provided correct responses, rather than simply adding 600 milliseconds to the error latencies. This approach better reflects the participants' response patterns and aligns with the concept employed by Jones et al. (2019). Additionally, we conducted a simple analysis to determine whether different age groups made more errors compared to others by conducting t-tests on accuracy scores; however, we found no significant differences (This has also been included in supplementary materials S4). Overall, the current approach of using the improved scoring algorithm, as described, and compared against other possible scoring mechanisms proposed by Greenwald, represents the most accurate way to analyse the IAT.

7. Are there additional modifications or refinements to the IAT scoring algorithm that could improve its validity in measuring implicit personality traits, particularly in diverse populations such as older adults or individuals with autism traits?

- The error correction applied in the scoring algorithm, similar to the approach by Jones et al. (2019), already addresses one way to enhance the scoring system. Work by Gonsalkorale et al. (2009) suggests people who are older might actually be better measured by the IAT (https://www.sciencedirect.com/science/article/abs/pii/S0022103108002370) . While a more refined scoring algorithm might be possible, it would require extensive testing. Additionally, some form of modeling approach could be considered; however, we strongly believe it is best to adhere to the traditional methods employed in the established IAT algorithms, as deviating from these practices may not be advisable.

Response to Reviewer 2

1. The description of the IAT task is not easy for people unfamiliar with the test to understand. Image examples are given, but word examples were only given in the text but not the figures. A couple examples of the blocks with corresponding image/word pairs would be helpful.

- This has been amended in the manuscript by including IAT block design as an example. Please see page 12, line 277, figure 2.

2. No example images for the Cambridge Face Memory task (CFMT) were shown. Were the composites all from female Caucasian, as in the IAT task? Or were they more diverse? Can this affect the interpretation on the results regarding the effect of face memory on implicit personality trait judgement?

- This is an excellent point. Given the CFMT is a well-established measure, example stimuli were not included in our study. Additionally, we don’t have copyright agreement for the image. Please see CFMT for original paper if you would like to view the images used in the study. The stimuli used in CFMT includes Caucasian young male faces. There is mixed evidence for gender differences in face memory performances (e.g. Scherf et al., 2017; Bobak et al., 2019). However, the effects are very minimal and not well established. As such, this should not affect the results of the current study. Female faces CFMT is not as robust as male CFMT. This still requires a lot of test re-test reliability. However, we will certainly consider using this in the future.

3. Regarding statistical tests, for either young or older adults in the IAT extraversion test, one-sample t-test was used for the D scores (Line 354), but when comparing between young and old, and Mann-Whitney (Line 368) was used. Were these two groups normally distributed or not?

- The data was normally distributed. An independent t-test results are included along with effect size estimates. Changes are reflected in the manuscript.

4. Line 358-360: “Young adults were faster and more accurate on trials where highly extraverted faces were paired with highly extraverted words, and on trials where lower trait faces were paired with lower trait words.” Need to clarify which part of the data support this claim (e.g. a significantly positive D score, etc). Related, an explanation on the interpretation of the D score would be helpful, such as what a non-zero score mean, in addition to defining the value mathematically.

- When IAT D scores are approximately 0, it denotes that there is not much difference in response latency between congruent (correct pairings) and incongruent (incorrect) conditions. Additional line as per suggestion, has been included on the amended manuscript on what positive D score means.

5. Was there a sex difference in any of the results, especially when only female composites were used?

- Although this wasn’t the main focus of the study, there were no gender differences across the IATs. This was tested using independent t-tests, yielding no significant differences in gender. This has now been included in the supplementary material Appendix S3.

6. For the neuroticism group, Line 205 indicates there were 120 young adults but in Table 5 the number is 118.

- Error amended. There are 120 young adults in the neuroticism

Attachment Submitted filename: Response to Reviewers.docx

# Peer review: Decision Letter 1

**DOI:** 10.1371/journal.pone.0322165.r004

**Authors:** Hosam Al-Samarraie

17 Mar 2025

Do young and older adult populations perform equivalently across different automatic face-trait judgements? Evidence for differential impacts of ageing.

PONE-D-24-06206R1

Dear Dr. Kannan,

We’re pleased to inform you that your manuscript has been judged scientifically suitable for publication and will be formally accepted for publication once it meets all outstanding technical requirements.

Within one week, you’ll receive an e-mail detailing the required amendments. When these have been addressed, you’ll receive a formal acceptance letter and your manuscript will be scheduled for publication.

An invoice will be generated when your article is formally accepted. Please note, if your institution has a publishing partnership with PLOS and your article meets the relevant criteria, all or part of your publication costs will be covered. Please make sure your user information is up-to-date by logging into Editorial Manager at Editorial Manager®  and clicking the ‘Update My Information' link at the top of the page. If you have any questions relating to publication charges, please contact our Author Billing department directly at authorbilling@plos.org.

If your institution or institutions have a press office, please notify them about your upcoming paper to help maximize its impact. If they’ll be preparing press materials, please inform our press team as soon as possible -- no later than 48 hours after receiving the formal acceptance. Your manuscript will remain under strict press embargo until 2 pm Eastern Time on the date of publication. For more information, please contact onepress@plos.org.

Kind regards,

Hosam Al-Samarraie

Academic Editor

PLOS ONE

Additional Editor Comments (optional):

Thank you for addressing all the comments in this revised version. 

Reviewers' comments:

Reviewer's Responses to Questions

**Comments to the Author**

1. If the authors have adequately addressed your comments raised in a previous round of review and you feel that this manuscript is now acceptable for publication, you may indicate that here to bypass the “Comments to the Author” section, enter your conflict of interest statement in the “Confidential to Editor” section, and submit your "Accept" recommendation.

Reviewer #1: All comments have been addressed

**********

2. Is the manuscript technically sound, and do the data support the conclusions?

The manuscript must describe a technically sound piece of scientific research with data that supports the conclusions. Experiments must have been conducted rigorously, with appropriate controls, replication, and sample sizes. The conclusions must be drawn appropriately based on the data presented. 

Reviewer #1: Yes

**********

3. Has the statistical analysis been performed appropriately and rigorously? 

Reviewer #1: Yes

**********

4. Have the authors made all data underlying the findings in their manuscript fully available?

The PLOS Data policy requires authors to make all data underlying the findings described in their manuscript fully available without restriction, with rare exception (please refer to the Data Availability Statement in the manuscript PDF file). The data should be provided as part of the manuscript or its supporting information, or deposited to a public repository. For example, in addition to summary statistics, the data points behind means, medians and variance measures should be available. If there are restrictions on publicly sharing data—e.g. participant privacy or use of data from a third party—those must be specified.

Reviewer #1: (No Response)

**********

5. Is the manuscript presented in an intelligible fashion and written in standard English?

PLOS ONE does not copyedit accepted manuscripts, so the language in submitted articles must be clear, correct, and unambiguous. Any typographical or grammatical errors should be corrected at revision, so please note any specific errors here.

Reviewer #1: (No Response)

**********

6. Review Comments to the Author

Please use the space provided to explain your answers to the questions above. You may also include additional comments for the author, including concerns about dual publication, research ethics, or publication ethics. (Please upload your review as an attachment if it exceeds 20,000 characters)

Reviewer #1: The Author has addressed and answered all the comments and concerned raised in prior review. The manuscript was revised accordingly.

**********

7. PLOS authors have the option to publish the peer review history of their article (what does this mean? ). If published, this will include your full peer review and any attached files.

If you choose “no”, your identity will remain anonymous but your review may still be made public.

**Do you want your identity to be public for this peer review?** For information about this choice, including consent withdrawal, please see our Privacy Policy .

Reviewer #1: No

**********

# Editor report: Acceptance letter

**DOI:** 10.1371/journal.pone.0322165.r005

**Authors:** Hosam Al-Samarraie

PONE-D-24-06206R1

PLOS ONE

Dear Dr. Tree,

I'm pleased to inform you that your manuscript has been deemed suitable for publication in PLOS ONE. Congratulations! Your manuscript is now being handed over to our production team.

At this stage, our production department will prepare your paper for publication. This includes ensuring the following:

* All references, tables, and figures are properly cited

* All relevant supporting information is included in the manuscript submission,

* There are no issues that prevent the paper from being properly typeset

If revisions are needed, the production department will contact you directly to resolve them. If no revisions are needed, you will receive an email when the publication date has been set. At this time, we do not offer pre-publication proofs to authors during production of the accepted work. Please keep in mind that we are working through a large volume of accepted articles, so please give us a few weeks to review your paper and let you know the next and final steps.

Lastly, if your institution or institutions have a press office, please let them know about your upcoming paper now to help maximize its impact. If they'll be preparing press materials, please inform our press team within the next 48 hours. Your manuscript will remain under strict press embargo until 2 pm Eastern Time on the date of publication. For more information, please contact onepress@plos.org.

If we can help with anything else, please email us at customercare@plos.org.

Thank you for submitting your work to PLOS ONE and supporting open access.

Kind regards,

PLOS ONE Editorial Office Staff

on behalf of

Dr Hosam Al-Samarraie

Academic Editor

PLOS ONE
